# Ribosome profiling reveals translation control as a key mechanism generating differential gene expression in *Trypanosoma cruzi*

**DOI:** 10.1186/s12864-015-1563-8

**Published:** 2015-06-09

**Authors:** Pablo Smircich, Guillermo Eastman, Saloe Bispo, María Ana Duhagon, Eloise P Guerra-Slompo, Beatriz Garat, Samuel Goldenberg, David J Munroe, Bruno Dallagiovanna, Fabiola Holetz, Jose R Sotelo-Silveira

**Affiliations:** Department of Genomics, Instituto de Investigaciones Biológicas Clemente Estable, Av. Italia 3318, Montevideo, CP 11600 Uruguay; Laboratory of Molecular Interactions, School of Sciences, Universidad de la República, Montevideo, Uruguay; Department of Genetics. School of Medicine, Universidad de la República, Montevideo, Uruguay; Laboratory of Gene Expression Regulation Studies Carlos Chagas Institute, FIOCRUZ, Curitiba, 81350-010 Brazil; Cancer Research Technology Program, Leidos Biomedical Research, Inc., Frederick National Laboratory for Cancer Research, Frederick, MD 21702 USA; Department of Cell and Molecular Biology, School of Sciences, Universidad de la Republica, Montevideo, Uruguay

**Keywords:** *Trypanosoma cruzi*, Neglected disease, Transcriptome, Ribosome profiling, Translatome, Translation regulation, Ribosomal proteins, Trans-sialidases

## Abstract

**Background:**

Due to the absence of transcription initiation regulation of protein coding genes transcribed by RNA polymerase II, posttranscriptional regulation is responsible for the majority of gene expression changes in trypanosomatids. Therefore, cataloging the abundance of mRNAs (transcriptome) and the level of their translation (translatome) is a key step to understand control of gene expression in these organisms.

**Results:**

Here we assess the extent of regulation of the transcriptome and the translatome in the Chagas disease causing agent, *Trypanosoma cruzi,* in both the non-infective (epimastigote) and infective (metacyclic trypomastigote) insect’s life stages using RNA-seq and ribosome profiling. The observed steady state transcript levels support constitutive transcription and maturation implying the existence of distinctive posttranscriptional regulatory mechanisms controlling gene expression levels at those parasite stages. Meanwhile, the downregulation of a large proportion of the translatome indicates a key role of translation control in differentiation into the infective form. The previously described proteomic data correlate better with the translatomes than with the transcriptomes and translational efficiency analysis shows a wide dynamic range, reinforcing the importance of translatability as a regulatory step. Translation efficiencies for protein families like ribosomal components are diminished while translation of the transialidase virulence factors is upregulated in the quiescent infective metacyclic trypomastigote stage.

**Conclusions:**

A large subset of genes is modulated at the translation level in two different stages of *Trypanosoma cruzi* life cycle. Translation upregulation of virulence factors and downregulation of ribosomal proteins indicates different degrees of control operating to prepare the parasite for an infective life form. Taking together our results show that translational regulation, in addition to regulation of steady state level of mRNA, is a major factor playing a role during the parasite differentiation.

**Electronic supplementary material:**

The online version of this article (doi:10.1186/s12864-015-1563-8) contains supplementary material, which is available to authorized users.

## Background

*Trypanosoma cruzi* is the causative agent of Chagas’ disease a serious ailment that affects millions of people in Latin America, against which there is no prevention, vaccine or effective chemotherapeutic agent [1].

*T. cruzi* has a complex life cycle characterized by several developmental forms present in vertebrate and invertebrate hosts. Replicative epimastigote and amastigote forms in arthropod and mammal hosts, respectively, alternate with the infective and non-proliferative metacyclic trypomastigotes in the insect vector and bloodstream trypomastigotes in the infected mammal [2]. The interchange between functionally and morphologically distinct forms implies tight control of gene expression during the life cycle of the parasite [3]. Understanding this comprehensive gene reprogramming, as well as their associated regulatory mechanisms, could contribute greatly to the control of Chagas’ disease.

Trypanosomatids probably belong to the earliest diverging branches of the eukaryotic lineage [4,5] and are characterized by their unique set of molecular characteristics. In *T. cruzi*, most genes are transcribed by RNA Polymerase II which generates polycistronic transcripts in a run-through fashion. Although transcription starts at defined locations, no sequence signals defining a classical eukaryotic promoter have been found at those sites [6-9]. In contrast to what occurs in bacterial operons, genes present in the same cistron are not functionally related and mature mRNA is obtained by trans-splicing and polyadenylation [10]. There is little evidence of transcriptional regulation for protein coding genes [11,12], however individual genes belonging to a common polycistronic unit show different expression patterns. In *T. cruzi* this was confirmed using microarrays [13]. Therefore, the control of gene expression has been thought to occur predominantly by posttranscriptional mechanisms [14]. In addition to regulation of mRNA turnover and protein degradation, early studies have recognized translation as an important regulatory step [3]. Single gene analyses have further confirmed this hypothesis [15-17], thus genome-wide translation studies are needed.

Next-generation sequencing (NGS) technologies have permitted not only the accurate determination of mRNA steady state levels but also the genome-wide analysis of processes such as transcription initiation, mRNA maturation, degradation and more recently translation. Specifically, deep sequencing of ribosome protected mRNA fragments, ribosome profiling, has provided a highly accurate measurement of the translation process *in vivo* [18], for a review see Ingolia 2014 [19]. Although NGS derived data for the related trypanosomatid parasite *Trypanosoma brucei* are accumulating [8,20-23], key differences in the biology and pathogenesis with *T. cruzi* clearly drive forward carrying out genome-wide approaches in this parasite. As reviewed in Kramer 2012 [14], remarkable differences include different life cycles (presenting *T. cruzi* an intracellular stage), vector transmission (salivaria or stercoraria), targeted host tissues (being *T. brucei* restricted to bloodstream), and immune evasion system (presenting *T. brucei* antigenic variation), and even molecular processes (such as absence of RNAi in *T. cruzi*).

In an effort to contribute to understand gene expression regulation processes occurring during the differentiation from the non-infective epimastigote (E) to the infective metacyclic trypomastigote forms (MT) we comprehensively monitored the steady state transcript abundances and translation profiles using RNA-seq and ribosome profiling. Our results strongly support previous indications of genome-wide constitutive transcription and uncover general pre mRNA maturation. In addition we reveal translation control as a key mechanism generating the gene expression changes that occur in *T. cruzi* differentiation.

## Results and discussion

### mRNA steady state levels support constitutive transcription and posttranscriptional regulation both in *T. cruzi* epimastigotes and metacyclic trypomastigotes

Ribosome profiling approaches require transcriptome determination in order to specifically estimate translational regulation. So, using RNA-seq we measured the steady state transcriptomes for both the epimastigote and metacyclic trypomastigote life cycle stages of *Trypanosoma cruzi.* The general procedure is shown in the Additional file 1. In brief, we sequenced biological triplicates of polyadenylated RNA from *T. cruzi* epimastigotes and *in vitro* differentiated metacyclic trypomastigotes by standard protocols in the SOLiD platform. In parallel, ribosome footprints, obtained in triplicates by digestion of polysomal fractions that were previously separated through sucrose cushions, were sequenced in the same platform for both stages. After quality filtering, reads from both were mapped to the *T. cruzi* reference genome and read counts per gene were calculated. Normalization was performed to account for sequencing depth and transcript length, resulting in an expression estimate for each gene (normalized reads per kilobase, nRPK, see Methods for further information).

**Figure 1:**
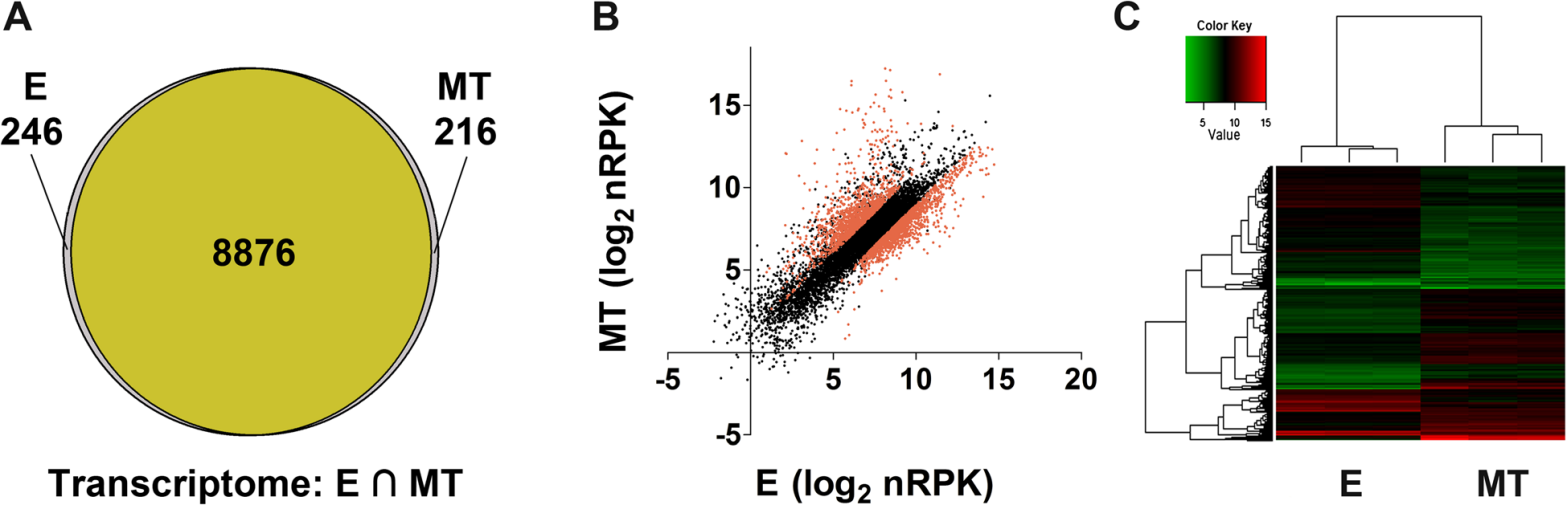
mRNA levels are regulated both in epimastigote (E) and metacyclic trypomastiogote (MT) life cycle stages. **(** **A** **)** Venn diagram indicating, at each stage, the number of detected genes (see Methods for detection criteria). The intersection is colored. **(** **B** **)** Scatter plot of the estimated expression levels as nRPK for both stages. Differentially expressed genes are shown in red (FC > 2, FDR < 0.05). **(** **C** **)** Heat map showing the variation of expression for the genes showing differences at the mRNA level at each stage.

Most of the 10600 annotated transcripts are detected in the mRNA fractions for the E (9122) and MT (9092) stages (using a detection cutoff of 15 normalized counts per gene), including a high number of them (8876, ≈95%) common to both transcriptomes (Figure 1A). These findings are in agreement with the hypothesis of constitutive transcription [13]. In addition, considering that the sequenced RNA sample is polyadenylated, these results also suggest constitutive RNA maturation. Nonetheless, one quarter (25.9% percent) of the transcripts detected showed at least a two-fold change in expression levels (FDR < 0.05) between the two developmental stages (Figure 1B and C, Additional file 2), supporting that posttranscriptional regulation is a determining factor to achieve the differential mRNA steady state levels.

### Metacyclic trypomastigotes translate fewer proteins than epimastigotes

To proceed to perform translation studies, genome-wide distribution of ribosome footprints (RFPs) mapping was assessed. In contrast to transcriptome derived reads, RFPs aligned mainly to annotated CDS segments while marginally to inter CDS regions (Additional file 3A). In addition, only a few genes lacking detectable transcripts were detected in the RFP fraction (0.9% and 1.4% for E and MT respectively). Furthermore, as ribosome translocation during translation should generate reads which are separated by three nucleotides, a periodicity of the mapping coordinate was expected for RFPs [18,24]. Although periodicity was marginal in MT translatome, E RFPs mapped more frequently to the first codon position with the second codon position being the less represented [18,24] (Additional file 3B). As expected, this three-nucleotide periodicity was not observed for the transcriptome data. Similar results for these quality control approaches were obtained by different authors [18,23,24]. Altogether, these observations support that RFPs reads are originated from the translating mRNA population.

**Figure 2:**
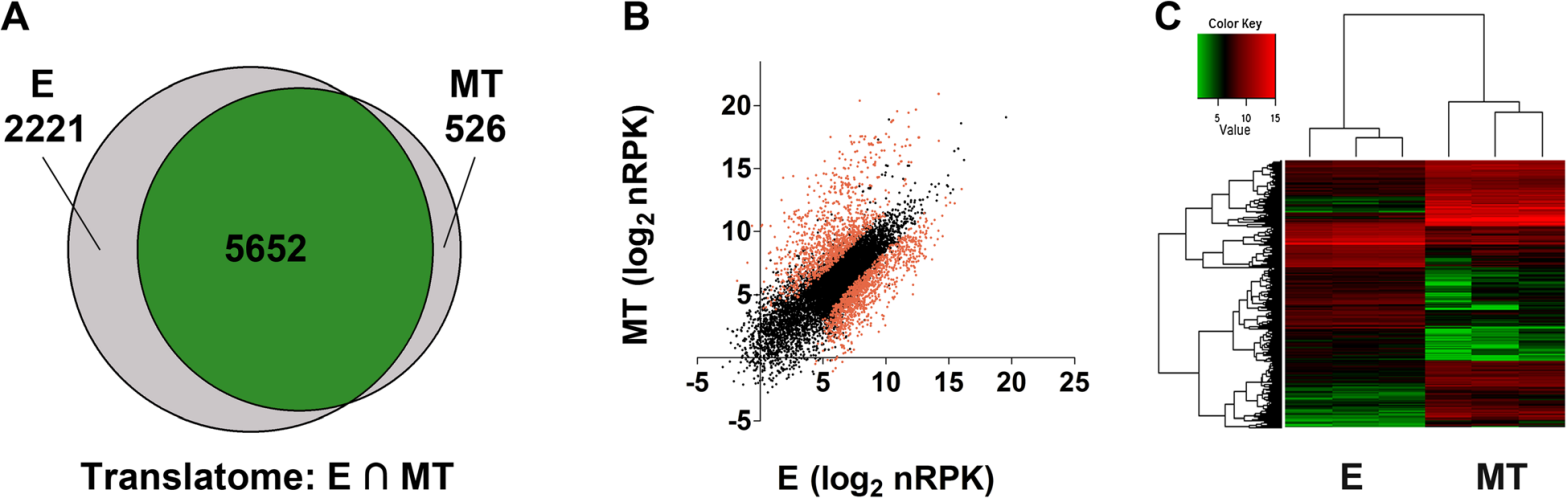
Translation is regulated in epimastigotes (E) and metacyclic trypomastigotes (MT). **(** **A** **)** Venn diagram indicating, at each stage, the number of detected genes (see Methods for detection criteria). The intersection is colored. **(** **B** **)** Scatter plot of the estimated translated levels as nRPK for both stages. Differentially expressed genes are shown in red (FC > 2, FDR < 0.05). **(** **C** **)** Heat map showing the variation of expression for the genes showing differences at the translation level at each stage.

**Figure 3:**
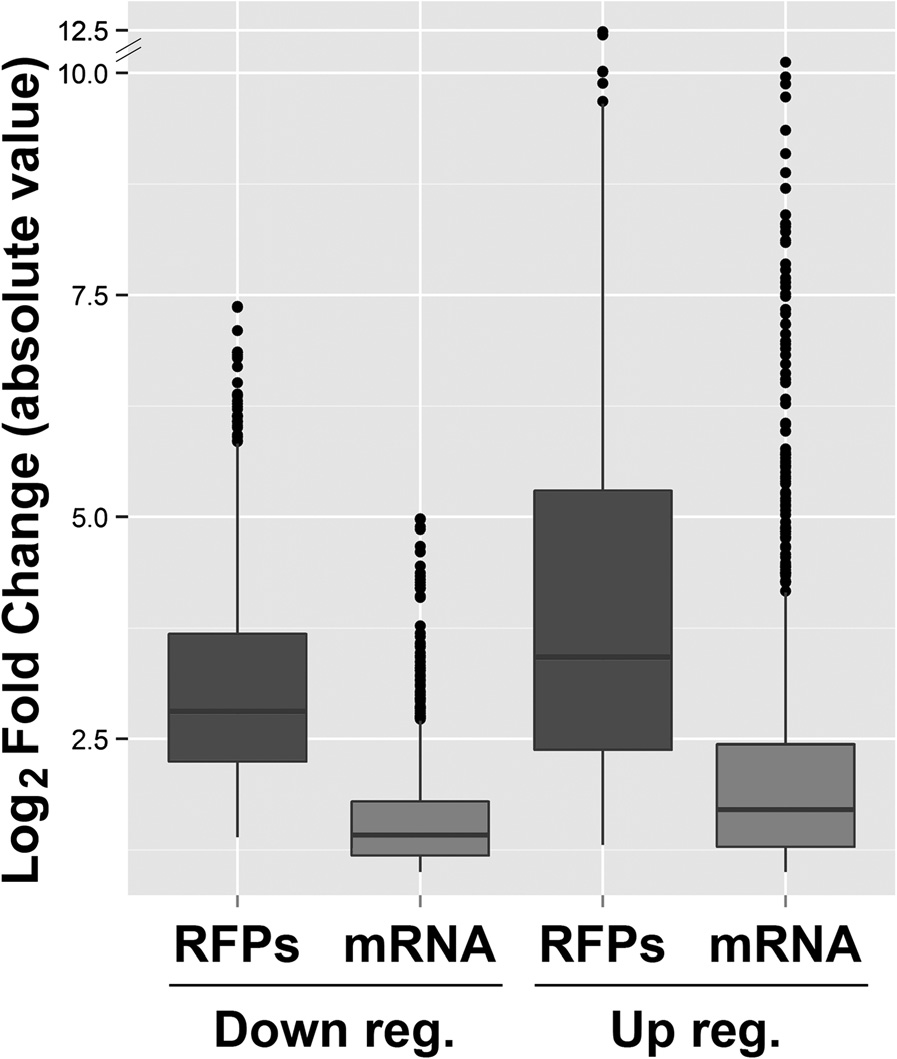
Regulation is higher at the translatome level than at the transcriptome level. Box plots of the fold change (MT/E expression estimates) distribution for regulated genes at the mRNA steady state level (light grey) or the translatome level (dark grey).

A broad picture of translation behavior at E and MT life cycle stages is shown in Figure 2. Although near 95% of the transcripts are common to E and MT (see above), only 67% of translated transcripts are common to both stages according to the same detection criteria used for the transcriptome data (15 normalized read counts per gene). Remarkably, this difference is explained by the absence of 2221 genes in MT translatomes (Figure 2A), with 80% of them having a repression fold change higher than 1.5. This finding reveals translation repression as a major regulatory mechanism in the infective form that could explain, at least partially, the proteome reduction previously reported for this stage [25]. Approximately a thousand genes belonging to the later category meet the criteria of FC < 0.5 and p-value < 0.05. This indicates that 10% of the annotated genes are significantly downregulated to levels that fall below our detection threshold at the translational level. In addition, the differentially translated genes between E and MT show a wider dispersion of the nRPK values than the one observed for the transcriptome (compare Figure 2B and C to Figure 1B and C, see also Figure 3 and the Additional file 4), reinforcing the relevance of translation control on gene expression regulation. It’s worth noting that the E to MT fold changes are higher in the translatome than in the transcriptome, resulting in a wider range of protein expression control (Figure 3). Interestingly, transcripts from gene families coding major metacyclic surface markers and proteins involved in the infection process account for almost half of the 526 genes detected only in the polysomes of the infective form (Additional file 5). Additional file 2 summarizes percentage changes corresponding to mRNA abundance and translation regulation. Table 1 shows the most up and downregulated protein coding genes (excluding pseudogenes) in the translatome fraction and Additional file 4 shows all the differentially regulated genes at the transcriptome and translatome levels.

**Table 1:** **Top 20 regulated protein coding genes in the RFP fraction**

**Upregulated**			
**Annotation**	**Feature ID**	**log2FC(MT/E)**	**FDR**
hypothetical protein	TcCLB.510323.60	11.93	5.55E-37
trans-sialidase putative	TcCLB.435601.10	11.10	3.43E-27
hypothetical protein conserved	TcCLB.506859.230	10.79	1.04E-32
hypothetical protein	TcCLB.509007.50	10.47	2.09E-32
receptor-type adenylate cyclase putative	TcCLB.428999.20	10.43	4.90E-09
trans-sialidase Group II putative	TcCLB.511585.230	10.42	1.04E-32
mucin-associated surface protein MASP putative	TcCLB.507957.320	10.38	1.05E-13
hypothetical protein	TcCLB.509433.10	10.02	3.30E-29
cyclin putative	TcCLB.509455.140	10.01	1.05E-25
DNA polymerase delta subunit 2 putative	TcCLB.509455.70	9.88	1.46E-29
hypothetical protein	TcCLB.507859.46	9.62	2.14E-08
hypothetical protein conserved	TcCLB.511545.170	9.61	1.75E-28
mucin-associated surface protein MASP putative	TcCLB.506599.100	9.58	2.06E-25
engulfment and cell motility domain 2 putative	TcCLB.509599.164	9.55	4.42E-11
hypothetical protein conserved	TcCLB.509769.20	9.26	9.15E-27
trans-sialidase putative	TcCLB.505363.19	9.13	9.80E-20
ATP-dependent DEAD/H RNA helicase putative	TcCLB.506777.10	9.07	2.26E-06
amino acid permease putative	TcCLB.509167.40	8.94	1.03E-25
protein kinase putative casein kinase I putative	TcCLB.510247.20	8.88	2.78E-23
phosphatidylinositol 3-kinase catalytic subunit putative	TcCLB.511709.19	8.86	1.63E-25
**Downregulated**			
**Annotation**	**Feature ID**	**log2FC(MT/E)**	**FDR**
40S ribosomal protein S33 putative	TcCLB.506413.30	NA	4.72E-09
hypothetical protein conserved	TcCLB.506659.35	NA	8.80E-06
hypothetical protein conserved	TcCLB.508207.54	NA	4.59E-03
hypothetical protein conserved	TcCLB.509599.120	NA	7.19E-04
hypothetical protein conserved	TcCLB.511527.82	NA	2.25E-02
anti-silencing protein ASF 1 putative	TcCLB.511417.100	NA	2.15E-03
hypothetical protein conserved	TcCLB.510515.120	NA	1.34E-02
hypothetical protein conserved	TcCLB.507611.50	NA	5.81E-03
hypothetical protein conserved	TcCLB.511529.50	NA	1.44E-03
RNA polymerase I	TcCLB.504041.4	NA	5.55E-03
hypothetical protein	TcCLB.504449.40	NA	1.16E-02
hypothetical protein	TcCLB.508277.310	NA	3.51E-02
hypothetical protein conserved	TcCLB.507631.10	NA	4.28E-02
60S ribosomal protein L37a putative	TcCLB.511145.46	−7.37	1.26E-04
kinetoplast-associated protein 3 KAP3	TcCLB.511529.80	−7.36	1.20E-12
nucleoside phosphorylase putative	TcCLB.506865.2	−7.10	2.16E-12
hypothetical protein conserved	TcCLB.511189.84	−6.86	2.50E-05
MP44 putative	TcCLB.506925.390	−6.85	1.55E-04
hypothetical protein conserved	TcCLB.511751.166	−6.83	1.10E-03
hypothetical protein conserved	TcCLB.510289.99	−6.81	1.85E-04

NA: genes with zero counts in the MT stage.

In summary, the regulation of translation greatly contributes to the expression differences between the metacyclic trypomastigote and epimastigote.

### Translatome expression values are a better proxy of protein levels than the transcriptome ones

**Figure 4:**
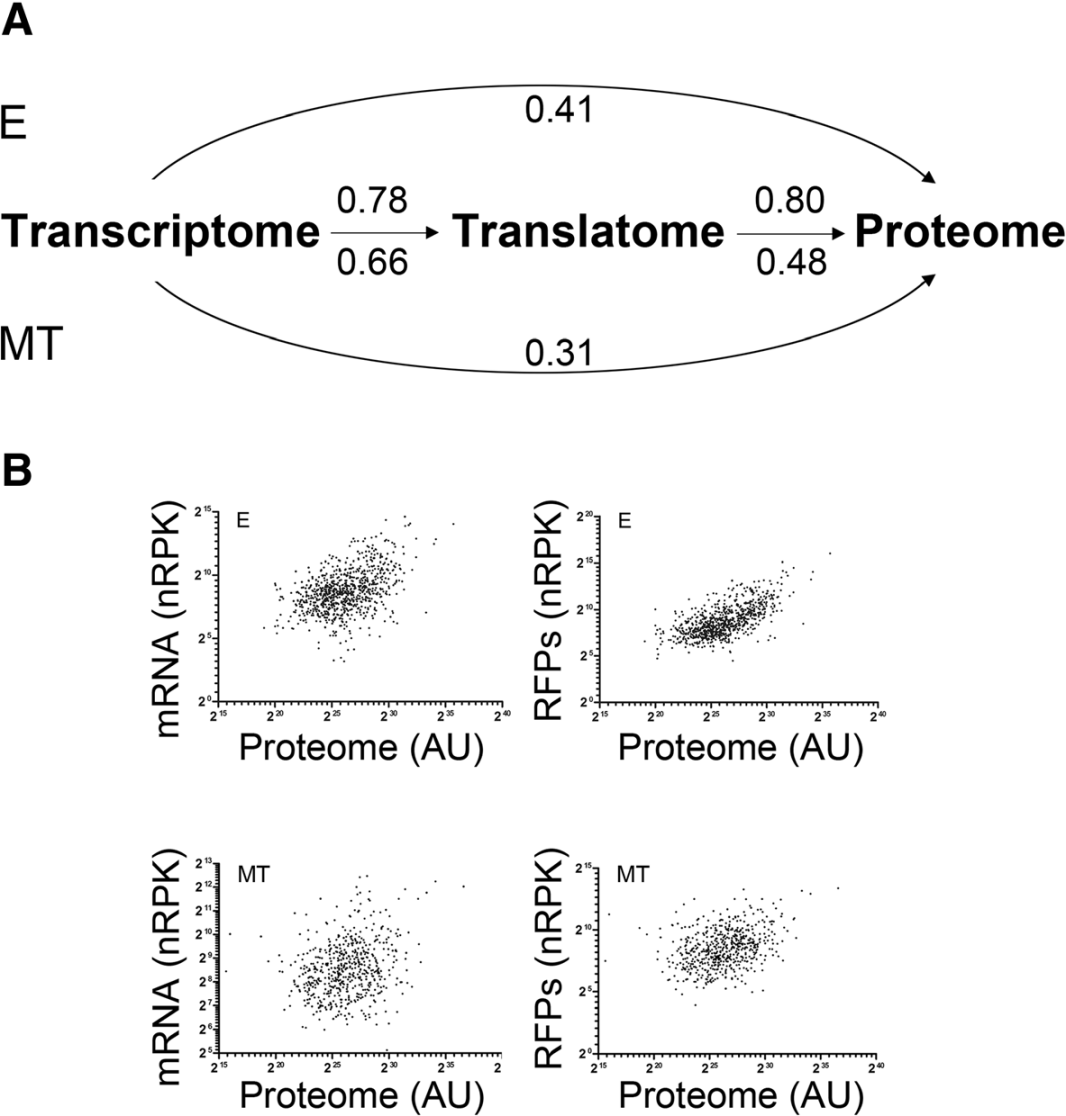
Translatome is better correlated with the proteome than the transcriptome. Inter sample correlations. **(** **A** **)** Pearson correlation coefficients for the different samples are shown. Upper values correspond to the ones observed in the E stage experiments. Lower values are as before for the MT stage. **(** **B** **)** Log-log scatter plots of the expression estimates in the E and MT stages. **Upper panel: Left**: Correlation of the proteome to the transcriptome in the E stage. **Right**: Correlation of the proteome to the translatome in the E stage. AU: Arbitrary units. **Lower panel:** Same as above for the MT stage.

Though in both E and MT life cycle stages the transcriptome and the translatome are well correlated (Pearson correlation coefficient r = 0.78 and 0.66 respectively, Additional file 6), we considered that translatome data, which represent the levels of translating RNAs, should be a better approximation of protein levels than transcriptomic data. Therefore, we decided to compare our translatome derived expression estimates to the available ones for quantitative proteomics in *T. cruzi* [26]. Figure 4 shows the correlation of the proteome to the transcriptome and the translatome for the E and MT stages. It can be clearly observed that translatome data are better correlated to protein expression than transcriptome levels. This is especially true for the epimastigote stage where the correlation coefficient between translatome and proteome goes up from a correlation coefficient of 0.41 for the transcriptome to 0.80 for the translatome. While an increase of the correlation for the latter is also observed in metacyclic trypomastigotes, it is not as significant (r = 0.31 and 0.48 for transcriptome and translatome to proteome respectively). No bias in the correlations was observed for the subset of the genes with proteome available data when comparing their transcriptome and translatome values (r = 0.76 and 0.63 for E and MT respectively, red dots in Additional file 6).

In summary, for the two life cycle stages analyzed, the translatome data are better correlated to proteomic data, reflecting the relevance of translational gene regulation and its contribution to the control of gene expression regulation during trypanosome development.

### Translation efficiency varies among the different genes and upon life cycle stages

**Figure 5:**
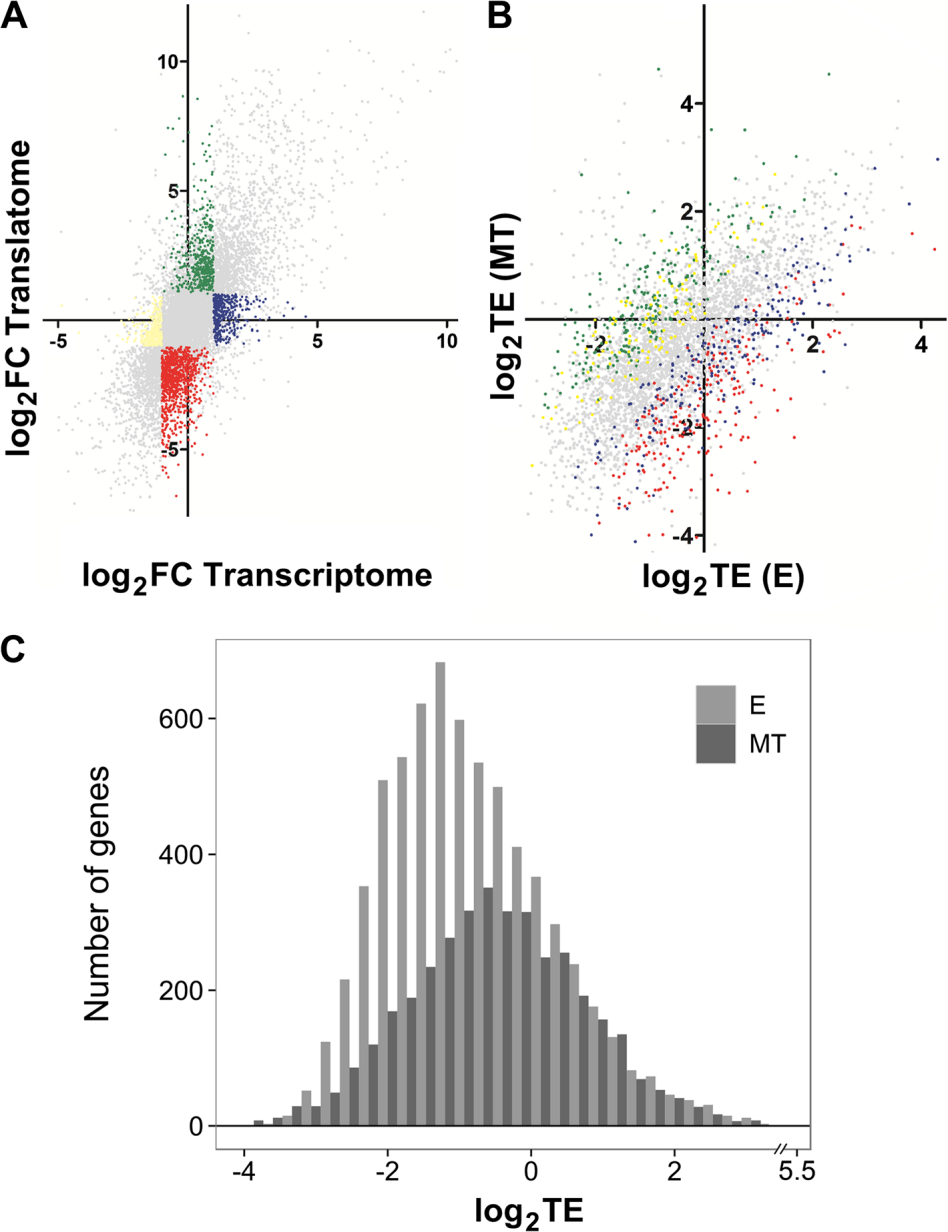
Translation efficiency (TE) varies between the epimastigote (E) and metacyclic trypomastigote (MT) *T. cruzi* stages. **Upper panel:** **(** **A** **)** Scatter plot of the fold change (MT/E expression estimates) in the translatome *vs* the transcriptome. **(** **B** **)** Scatter plot of the TE (RFP/Total RNA expression estimates) in the MT *vs* the E stage for genes detected in all samples. Genes exhibiting non proportional changes (FC > 2, FDR < 0.05) are colored. **Lower panel: (** **C** **)** TE histograms for epimastigotes (light grey) and metacyclic trypomastigotes (dark grey). Median efficiency values are 0.51 and 0.69 respectively.

In an effort to determine the contribution of the steady state transcript levels and the extent of their translation upon differentiation, we calculated the MT relative to E expression levels for each gene both in the transcriptome and the translatome. We found that a high number of CDS exhibit non proportional changes when a FC > 2, p-value < 0.05 is considered (colored genes in Figure 5A). Translation efficiency (TE) is defined as the number of footprints per transcript and it gives an idea of the ribosome occupancy per messenger molecule. As previously reported for other organisms including *T. brucei* [18,23,27-29], *T. cruzi* TE is highly variable for the different mRNAs in both life stages (Figure 5B and C), ranging from values close to 0 to values of 40. We found that numerous genes change their TE upon differentiation. Using a two-fold change as a determination of differential expression, 643 genes are regulated exclusively at the level of translation upon differentiation (Figure 5A, green and red dots). These results further support the importance of translation in the regulation of stage-specific gene expression.

**Figure 6:**
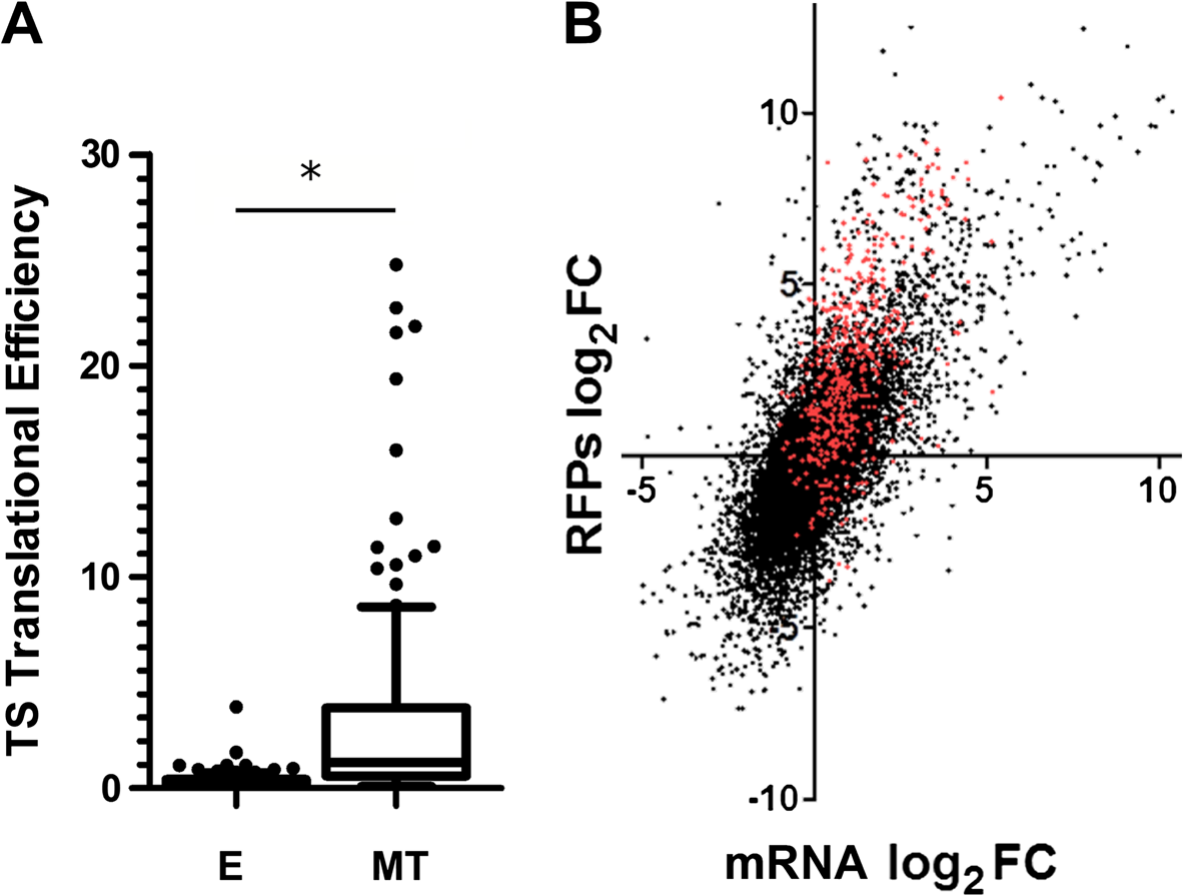
Trans-sialidase (TS) family genes increase their TE upon differentiation. Expression profiles for the TS genes in *T. cruzi* epimastigotes (E) and metacyclic trypomastigotes (MT) are shown. **(** **A** **)** Box plots of the TS family translational efficiency in E and MT. Statistically significant differences among populations are indicated by asterisks (Wilcoxon p < 0.05). **(** **B** **)** Scatter plot of the fold change (MT/E expression estimates) in the translatome *vs* the transcriptome. TS genes are shown in red.

In the MT stage, genes coding for members of the trans-sialidase (TS) superfamily are the most overrepresented among the ones with an efficiency FC (MT/E) higher than 2 (Additional file 7A). Actually, when the genes with the 1% highest TE in this stage (TE > 7.3) were analyzed for overrepresentation of Gene Ontology terms (GO analysis), only this family showed statistically significant values (Additional file 8, see Methods for details on the functional annotation procedure). They are also the second most significant among the ones that increase their translation (but not the amount of mRNA, i.e. genes increasing their TE) in the MT stage (green dots in Figure 5 and Additional file 9A). Indeed, the TE of this family in the MT stage is significantly higher than in the E stage (Figure 6A). As can be observed in Figure 6B, the fold change is also positive for the mRNAs of many TS family members. Furthermore, while the translation levels of the TS family is significantly lower in the E stage when compared to the rest of the genes, in the MT stage the behavior is reversed explaining the high difference in TE between the stages (Additional file 10). The other group of genes that is overrepresented among the ones which only increase their translation levels encode for proteases (Additional file 9A). Manual inspection of the involved genes reveals that they mainly encode isoforms of the gp63 surface metalloproteases that have been recognized as important for host-cell infection by trypomastigotes [30]. GO analysis of the genes that have both an increased translation and mRNA steady state level in the MT stage, again show enrichment in TS family members (Additional file 9B). Several specific members of this superfamily have been shown to have key functions during host invasion. One of these well studied proteins is the adhesion molecule gp82 [31]. Our data show that members of this family increases an average of 30 fold its translational efficiency after differentiation to the MT stage (Additional file 11) in accordance with previous reports uncovering polysomal mobilization as a control step of its expression [32]. Other members shown to be relevant for parasite survival upon infection (as CRP or GP85, see genes TcCLB.511129.40, TcCLB.511911.60 and TcCLB.506455.30 in the Additional file 4), are also overexpressed in MT [33,34]. Interestingly, other genes coding for proteins related to specific cellular processes that have been previously recognized as upregulated during transition to the MT stage at the proteomic level (i.e. proteins related to cytoskeleton and RNA binding proteins) [26] are upregulated in the translatome according to our GO term enrichment analysis (Additional file 9B). This further supports the reliability of our approach and the close relationship observed between the translatome and the proteome.

**Figure 7:**
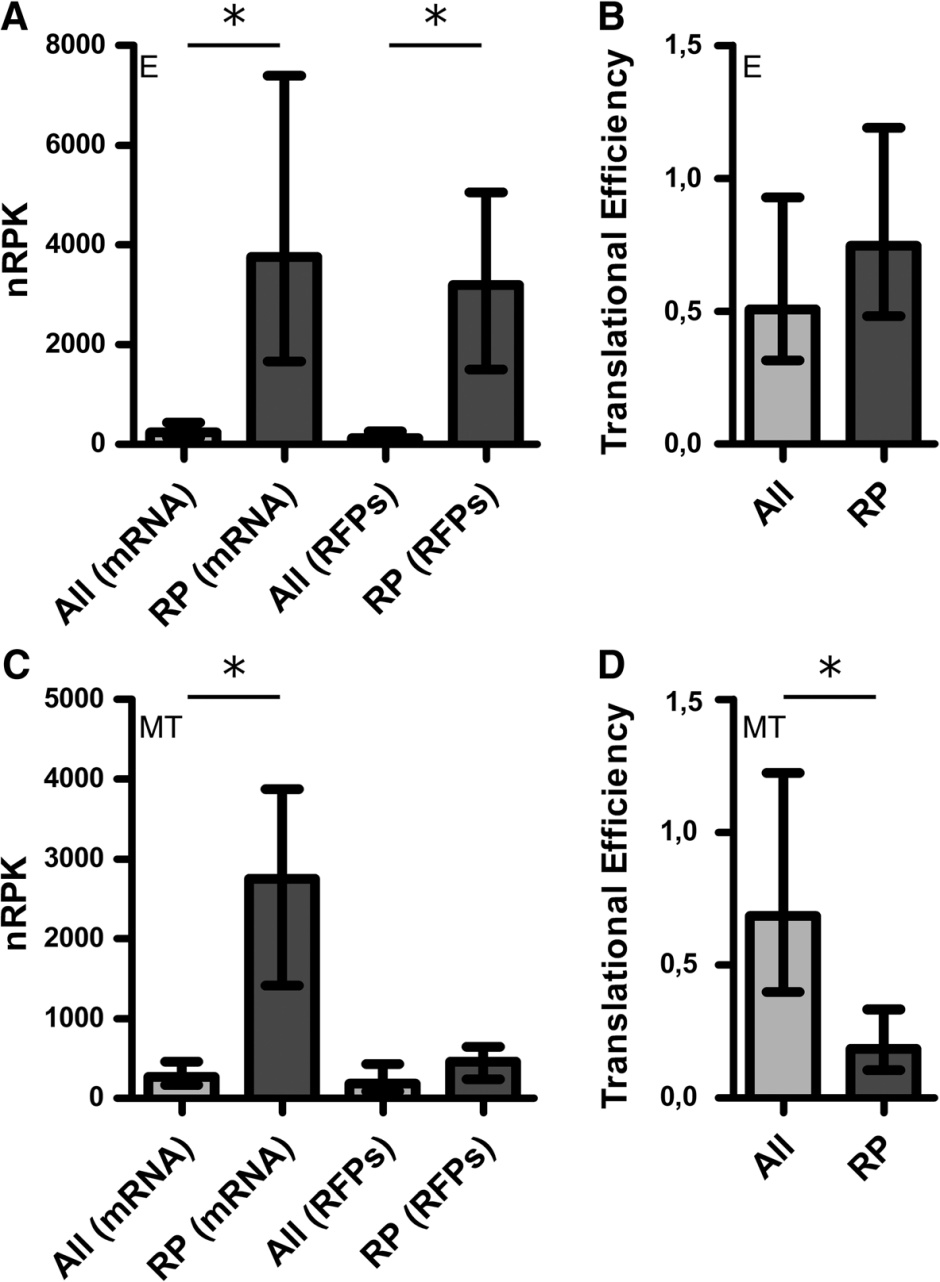
Ribosomal proteins (RP) genes decrease their TE upon differentiation. Expression profiles for the RP genes are shown in *T. cruzi* epimastigotes (E) and metacyclic trypomastigotes (MT). **Upper panel: (A)** Bar plot of the E stage transcriptome and translatome levels for the RP and for all *T. cruzi* genes. Each bar marks the population median while the whiskers represent the interquartile range. **(B)** Bar plot of the translation efficiency (TE) for the RP and for all *T. cruzi* genes. Statistically significant differences among populations are indicated by asterisks (Wilcoxon p < 0.05). **Lower panel: (C)** and **(D)** are the same type of bar plots as A and B respectively, but for the MT stage. Note the decrease in translation efficiency of RP **(D)** as a result of a major decrease in RP translation **(C)**. Y-axis scales in figures A and C are different.

On the other hand, in the MT stage, the genes coding for ribosomal proteins (RP) are the most overrepresented among the ones with an efficiency FC (MT/E) lower than 0.5 (Additional file 7B) in accordance with previous proteomic observations [25]. Our data show that this downregulation is mainly derived from the low translation levels of the RP genes in the MT compared to the E stage, since similar levels of steady state mRNA are found in both stages (Figure 7A and C). Furthermore, in the MT stage the TE of this group of genes is significantly lower than the TE for all the genes (RP TE median = 0.19 *vs* genome TE median = 0.69, Wilcoxon p < 0.05, Figure 7D).We also found that the gene encoding for RNA polymerase I is downregulated in the MT stage where no RFPs where detected (Additional file 4), suggesting a possible reduction of ribosomal RNA synthesis consistent with the downregulation of ribosomal protein production. Accordingly, GO analysis on the downregulated genes in the MT translatome, shows that gene families related to protein synthesis are significantly enriched (Additional file 12). Interestingly, genes coding for enzymes involved in the synthesis of hypusine are also downregulated in MT translatome (Additional file 12). This amino acid, which is found in all eukaryotes, is essential for the function of the eIF5A translation factor where it is post translationally synthesized from a lysine residue [35]. The eIF5A factor has been characterized in other models and, nowadays, it is recognized as a regulator of translation elongation involved in cell cycle progression [36]. Indeed, previous work in *T. cruzi* suggested that the expression levels and post translational modifications of this translation factor controls the cell proliferation rates and protein synthesis [37]. Overexpression of the factor in epimastigotes increases proliferation while in the MT stage the protein levels show a decrease [37]. The eIF5A genes are significantly downregulated in the the MT stage translatome (see IDs TcCLB.506925.120 and TcCLB.506925.130 in Additional file 4) which correlates with the observed downregulation of the hypusine addition in the non-replicative stage (Additional file 12). Another cell cycle related protein downregulated in the MT translatome stage is the cyclin CYC2 (TcCLB.507089.260) (Additional file 4). All these findings are in agreement with the quiescent characteristic of this stage and the downregulation of the translated proteins (Figure 2A). Further inspection of the identity of the stage specific regulated genes may yield more insights on the biology of the studied process. For example, we noticed the downregulation in the MT of the *T. cruzi* homolog of the Anti-Silencing Function protein (ASF1) (Additional file 4). This protein acts as a nucleosome assembly factor and as such would not be necessary in the non replicative infective stage [38].

Overall, these results highlight the relevance of translation efficiency allowing the rapid changes in gene expression necessary for differentiation.

## Conclusions

*Trypanosoma cruzi* has enigmatic gene expression control mechanisms that new *in-masse* approaches are beginning to unravel [13,25,26,39-42]. Where and how the final protein content is regulated is a matter of intense research. It is generally accepted that in the kinetoplastids, regulation is mainly posttranscriptional [12,43], being stability of mRNA and its translatability key steps and target of largely unknown regulation pathways. In the present manuscript, we aimed to characterize at a genome-wide level the steady state transcript levels and the extent of protein translation regulation using RNA-seq and ribosome footprinting in two *T. cruzi* life cycle stages, i.e. epimastigotes and metacyclic trypomastigotes. These two forms occur in the insect vector and correspond to the replicative, thus proliferating form, and the non-replicative and infective form, respectively.

Although a microarray based relative transcriptome is available for the comparative analysis of expression among the four *T. cruzi* life cycle stages [13], no RNA-seq data from *T. cruzi* have been reported so far. We found that both the E and MT stages transcribe most of the genes encoded in the *T. cruzi* genome (approx. 86.1% and 85.8% respectively), being almost all common to both stages (approx. 98%) (Figure 1 and Additional file 2). Our results not only support the accepted view of constitutive transcription but also show the existence of global mRNA maturation as implied by the detection of almost all the transcripts as poly(A)+ mRNA. However, 30% of the genes show significant differences in the mRNA steady state levels that, in the context of absence of transcriptional initiation control, can only be accounted by differential mRNA stability. The extensive transcript detection implies that mRNA decay may not be sufficient to achieve precise protein levels pointing out the need of additional regulatory mechanisms.

To investigate the degree of protein synthesis regulation in this parasite we performed ribosome profiling (Figure 2), an approach pioneered by the group of Weissman, that has been used very recently to improve the estimation of genome-wide protein synthesis in several eukaryotes (reviewed in [19], and references therein). In our hands, this technique allowed to determine protein synthesis levels in a proportion of genes that is three times greater than any other proteomic effort published in *T.cruzi* [25,26,42]. Interestingly, much more genes encoded in the *T. cruzi* genome are translated in the epimastigote (approx. 74%) than in the metacyclic trypomastigote (approx. 58%) stage (Figure 2). The translatome difference between the two life cycle stages is more pronounced than the one observed for the steady state levels of transcripts (Figure 1). The small translatome observed in the MT stage can be mainly explained by specific inhibition of translation of a significant percentage of mRNAs but reduction of assembled ribosomes could also be a contributing factor.

Recent quantitative proteomics in *T. brucei* has shown that the transcriptome and the proteome positively correlate [44]. Our data are in good accordance with this assertion, but protein translation rates derived from ribosome footprints correlate much better with the parasite proteome than transcriptome (Figure 4). When fold change analysis (MT over E) is studied, a wider spectrum of values for the translatome than the transcriptome is observed (Figure 3). This suggests that the translation process has broader capabilities for regulation than modification of mRNA steady state levels in this parasite. Although additional posttranslational regulatory steps are certainly operating both in E and MT stages, the low correlation of translatome *vs* proteome observed in MTs (Figure 4), suggests that such processes may be particularly active in this life cycle stage. For instance, regulation of the protein stabilization/degradation or ribosomal stalling during translation (which would produce RFPs but not functional protein product), could explain the lower correlation observed. These issues should be specifically addressed in further studies. The broader regulation that can be achieved with the observed translational regulation may be especially appropriate to generate rapid responses to the changing environment, from the vector’s gut to the mammalian host, affecting the infective MT stage.

In agreement with the recently described data for the closely related parasite *T. brucei* [23], in the present study we have observed large differences in translation efficiency among transcripts in the same life cycle stage and between the same transcript in the two life cycle stages (Figure 5). Thus, the regulation of translation efficiency constitutes a means to rapidly adjust the yield of specific protein products from the available mRNA steady state levels. We have focused on the conspicuous changes of translation efficiency of members of the trans-sialidases family (Figure 6). This large gene family of virulence factors, responsible for transferring sialyl residues from the host, are membrane proteins with an active role in infectivity and therefore, a high number of the family members are expressed in the MT stage [25,45,46]. On the other hand, the genes coding for ribosomal proteins also caught our attention because of the striking low TE exhibited in the MT stage (Figure 7). This family of proteins has been comprehensively studied in *T. cruzi* using data mining and mass spectrometry of purified epimastigote ribosomes [47]. This finding is consistent with the reduction in ribosome protein content previously reported for this non-replicative life cycle stage [25,26]. Thus, these gene families which encode principal actors defining major distinctive characteristics of the MT (a more quiescent stage mainly specialized in host cell invasion) undergo prominent changes of TE in the transition from E to MT. These results further support translational efficiency control as a key mean to achieve stage-specific gene expression regulation.

Interestingly, pseudogenes are detected in the transcriptome and the translatome of both *T. cruzi* stages. This is unlikely to be caused by misplacement of the reads coming from the parental gene, as the observation also holds when only single match reads are considered. Not only these sequences are detected but some of them are differentially purified both in the poly(A)+ mRNA and the RFP fractions of both stages (Additional file 4). Pseudogene transcription is nowadays widely accepted [48], and it has been demonstrated that these transcripts can be functional, in many cases controlling the expression of their parental gene [48]. More intriguingly, our data also shows evidence of pseudogene transcripts in the polysomal fractions. The potential of pseudogene derived transcripts to be translated has been poorly studied in the literature so far. There are some reports in other organisms showing that the short peptides resulting from this process exist and can produce phenotypic outcomes [49,50]. The existence of pseudogene expression in trypanosomes is an interesting finding, placing the analysis of their functional role as an issue that should be addressed in further studies.

In conclusion, the data here presented, generated from the non infective epimastigote and infective metacyclic trypomastigote *T. cruzi* life cycle stages, provide a comprehensive picture of the mRNA steady state level and their translation capability at both life cycle stages. Our results not only show that the mechanisms establishing mRNA steady state and translation levels are likely acting synergistically, but also point out to translation efficiency as an important intra- and inter-stage posttranscriptional regulatory program, remarkably active in the control of virulence factor expression in the insect infective forms.

## Methods

### Parasites

Epimastigotes of *T. cruzi* Dm28c strain [51] were cultured at 28°C in liver infusion tryptose (LIT) medium supplemented with 10% bovine fetal serum. The culture was initiated by adding 1 × 10^6^ cells mL^−1^ and the exponentially growing epimastigotes with less than 0.1% of metacyclic cells were obtained from three-day culture (density of 3 × 10^7^ epimastigotes ml^−1^). Three biological replicates with 2.5 × 10^9^ epimastigotes each were used. Metacyclic trypomastigotes were obtained as previously described [51,52]. Briefly, epimastigotes in the late exponential growth phase from five-day culture (density of 5–6 × 10^7^ parasites ml^−1^) were harvested by centrifugation at 7000 × g for 5 min at 25°C and subjected to nutritional stress for 2 h at 28°C in TAU medium (190 mM NaCl, 17 mM KCl, 2 mM MgCl2, 2 mM CaCl2, 8 mM phosphate buffer pH 6.0) at a density of 5 × 10^8^ parasites ml^−1^. The epimastigotes were subsequently used to inoculate cell culture flasks containing TAU3AAG (TAU supplemented with 50 mM sodium glutamate, 10 mM L-proline, 2 mM sodium aspartate and 10 mM glucose) at a density of 5 × 10^6^ cells mL^−1^ at 28°C. Metacyclic trypomastigotes were purified by DEAE-51 chromatography from the TAU3AAG culture supernatant after 72 h of incubation. Three biological replicates of 5 × 10^9^ parasites each with greater than 99% metacyclic cells were used.

### Library preparation and sequencing

Messenger RNA was purified using poly(A)+ mRNA selection, and sequenced using standard SOLiD RNA-seq procedures. Ribosome protected footprints were generated through nuclease treatment of cell extract in the presence of cycloheximide. The drug was added to a concentration of 100 μg/ml and incubated for 10 minutes at 28°C, and was present at this concentration in all downstream steps according Ingolia and cols. with minor modifications [18]. The polysomes were isolated through a sucrose cushion, under conditions previously established to enrich in polysomes [53,54], and the polysome enriched fraction was digested with RNAse. Treated RNA was extracted and ribosome-protected fragments (aprox. 30 nt) were separated and purified through FlashPAGE™ electrophoresis as previously described [29,18]. The experiments were performed in triplicate and the RFPs and poly(A)+ mRNA mRNA fraction was analyzed by deep sequencing on the Life Technologies SOLiD4 equipment (high throughput sequencing facility RPT01G PDTIS/Carlos Chagas Institute - Fiocruz Parana). Fragmented poly(A)+ mRNA was prepared from the same biological sample used to prepare RFP libraries. Raw sequence data was submitted to SRA [SRA: PRJNA260933].

### Sequence read processing, alignment, normalization and comparative analysis

Read trimming was performed using CLC Genomics Workbench 6.5 (CLC) with Q phred score larger than 13. A range between 25 and 40 nt was selected for the footprints lengths, while 18 to 50 nt was set for the transcriptome reads (see Additional file 13A for trimming statistics). Reads passing trimming criteria were analyzed using standard RNA-Seq protocols implemented in CLC. *T. cruzi* CL Brener Esmeraldo-Like annotated transcripts V5.0 was used as reference ("http://tritrypdb.org/"). Alignment settings for color space reads were the following: maximum number of mismatches: 2; minimum length fraction: 0.9; minimum similarity fraction: 0.8 and maximum number of hits for a read: 10 (see Additional file 13A for mapping statistics). For further analysis read counts were used as input in the DESeq package implemented in the R statistical environment [55]. Using this package, the six transcriptome samples (three replicates for each stage) where normalized against each other to account for the differences in sequencing depth; the same strategy was carried out independently for the six translatome samples. After normalization, replicate variability was assessed (Additional file 13B). The normalized read counts were divided by transcript length to obtain an expression estimate (nRPK). Differential expression across stages was assessed with the DESeq package, setting a fold change > 2 and a FDR < 0.05 to define differential genes. Genes were considered to be detected if a minimum of 15 DESeq normalized counts were mapped in each replicate. Similar to [18], a inter replica variation index (IRI) for each gene was calculated (standard deviation divided by the sum of the mapped reads in the replicates) and a cutoff value of 0.2 was set as gene inclusion criteria for the rest of the presented analysis (Additional file 9C). Heatmaps were constructed with the heatmap.2 R package using default parameters for distance and clustering calculations.

Independent experimental verification of the expression levels obtained in our transcriptome and translatome experiments was performed for a set of differentially expressed *T. cruzi* genes. Specific primers were designed for genes: 40S ribosomal protein TcRPS12 TcCLB.508551.20 (forward 5’TGCGAAGACGAGGAGTACAA3’, reverse 5’GCCACACACGAGCACTTAAA3’), TcS25 TcCLB.503907.10 (forward 5’AAAAGGGTCGGCTTCATCTT3’, reverse 5’CCGTCATCACCCTTCTTGTT3’), and trans-sialidase TcGP82 TcCLB.510307.230 (forward 5’AGAGAGAGTGAGCGGCAGAG3’, reverse 5’TGGAGTACCTCCACCTTTCG3’). RT-PCR was carried out from ribosome-free, monosomal and polysomal fractions extracted from both epimastigotes and metacyclic trypomastigotes of the Dm20c strain.

Sucrose density gradient of epimastigotes and metacyclic trypomastigotes extracts were prepared as previously described [53,54] and RNA was extracted for each fraction. PCR products were analyzed in 2% TBE-agarose gels and band density was calculated using the ImageJ software (Additional file 14).

Quantitative proteomic data were obtained from a label free MS-based approach [26].

### Functional annotation of gene lists

To categorize gene lists into overrepresented functional related groups, DAVID (Database for Annotation, Visualization and Integrated Discovery, version 6.7) functional annotation clustering tool was used [56]. Groups with an “enrichment score” (ES) > 1.3, (defined as the minus logarithm of the geometric median of p values) were considered significant [57].

### Availability of supporting data

The data sets supporting the results of this article are available in the Sequence Read Archive repository, Project ID: PRJNA260933.

## Additional files

Additional file 1:
**Diagram showing the main steps of the experimental design and data analysis.**
Additional file 2:
**Number of genes present in** ***T. cruzi*** **transcriptome and translatome and their inter stage variation.**
Additional file 3:
**Ribosome footprints originate from translation activity.** (*A*) Mapping characteristics of the reads obtained in the transcriptome (upper panel) and in the translatome studies (lower panel). A fragment of chromosome 12 (from approx. 395,000 to 400,000bp) is shown. CDSs in the region are represented as green arrows. (*B*) Mapping periodicity in *T. cruzi* epimastigotes. Bars represent the percentage of the reads that have their 5´ end mapping to each reading frame (see Materials and Methods). Translatome (dark grey) and transcriptome (light grey) mapping periodicity are shown. Additional file 4:
**Differentially expressed genes in the transcriptome and translatome fractions.**
Additional file 5:
**Lists of genes detected only in the MT stage translatome.**
Additional file 6:
**Transcriptome-translatome correlations.** Log-log scatter plot of the estimated expression levels as nRPK. The subset of genes detected in the proteomic studies are shown in red. (*A*) E: epimastigotes. Pearson correlations of 0.78 and 0.76 were calculated for all genes and for the proteome detected genes respectively. (*B*) MT: metacyclic trypomastigote. Pearson correlations of 0.66 and 0.63 were calculated for all genes and for the proteome detected genes respectively. Additional file 7:
**DAVID functional annotation clustering result for genes having a FC>2 (A) and a FC<0.5 (B) in their TE after differentiation.**
Additional file 8:
**DAVID functional annotation clustering result for the genes with the highest translational efficiency in** ***T. cruzi*** **metacyclic trypomastigotes (MT).**
Additional file 9:
**DAVID functional annotation clustering result for the genes increasing their translation after** ***T. cruzi*** **epimastigote to metacyclic trypomastigote differentiation.**
Additional file 10:
**Trans-sialidase (TS) family gene expression profile in** ***T. cruzi*** **epimastigotes (E) and metacyclic trypomastigotes (MT).** Comparison of translational efficiency for the TS family genes with the rest of the genes for the E (*A*) and MT (*B*) stages. Y-axis scales in figures A and B are different. Additional file 11:
**Expression analysis in epimastigotes and metacyclic trypomastigotes stages for the trans-sialidase gp82 coding genes.**
Additional file 12:
**DAVID functional annotation clustering result for the genes decreasing their translation after** ***T. cruzi*** **epimastigote to metacyclic trypomastigote differentiation.**
Additional file 13:
**Data filtering and assessment of replicates consistency.** (*A*) Trimming and mapping statistics for all the replicates of the transcriptome (mRNA) and translatome (RFPs) data. (*B*) Left panel: Heatmap of the distances for the different samples and replicates. Middle panel: 2D plot of the 2 first components of the principal component analysis for the different samples. Right panel: Table showing the Pearson correlation coefficients for the biological replicates. (*C*) Reproducibility across different mapping densities. Genes were binned by the number of mapped reads (50 reads per window). A boxplot for the distribution of the reproducibility index (IRI = σ/ ∑ TGR) was constructed for each bin and ordered by the number of mapped reads per gene (TGR, total gene reads) for the four samples studied. Additional file 14:
**Independent experimental verification of gene expression levels.** (*A*) Upper panel: result of RT-PCR experiments for ribosome-free mRNA (lane 1), monosomal (lane 2) and polysomal fractions (lane 3) for the selected genes. Lower panel: Polisome profiles obtained by sucrose gradients separation for each stage. The image indicates the span of the 3 fractions analyzed using horizontal lines. (*B*) Table showing the fold change in translation efficiency (metacyclic trypomastigote divided by epimastigote values) as assessed by both the above RT-PCR experiments (column 3) and by ribosome profiling (column 4). Translation efficiency for the RT-PCR experiments was calculated by first quantifying band density (ImageJ) and then dividing the value obtained in the polysome fraction by the average value obtained in free mRNA and monosome fractions.
