# Supplementary figures and images for: Ribosome profiling reveals translation control as a key mechanism generating differential gene expression in Trypanosoma cruzi
Source: BMC Genomics. 2015 Jun 9;16(1):443. doi: 10.1186/s12864-015-1563-8 (PMC4460968; doi:10.1186/s12864-015-1563-8)

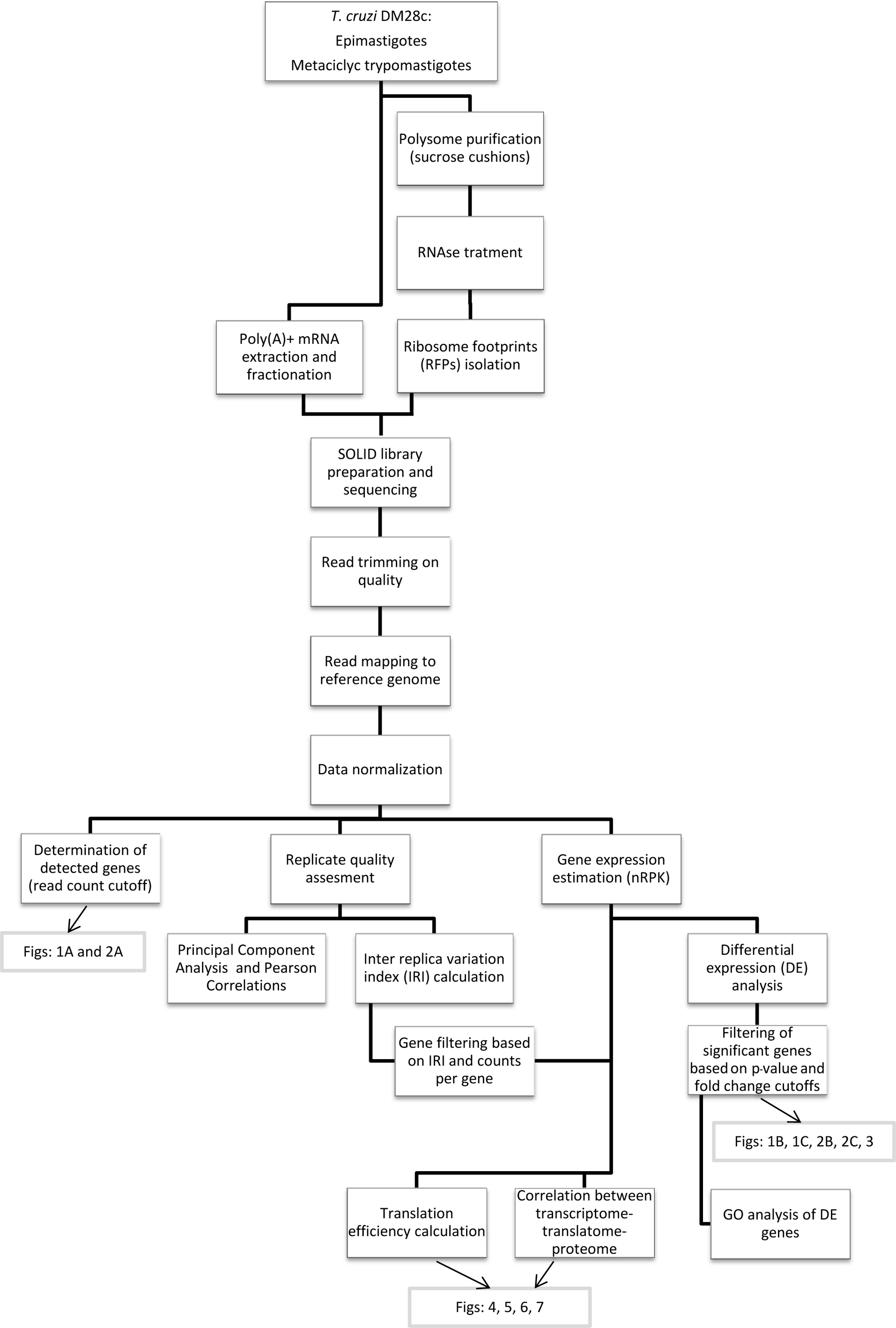

Supplement: Additional file 1: — Diagram showing the main steps of the experimental design and data analysis. [file 12864_2015_1563_MOESM1_ESM.tiff]

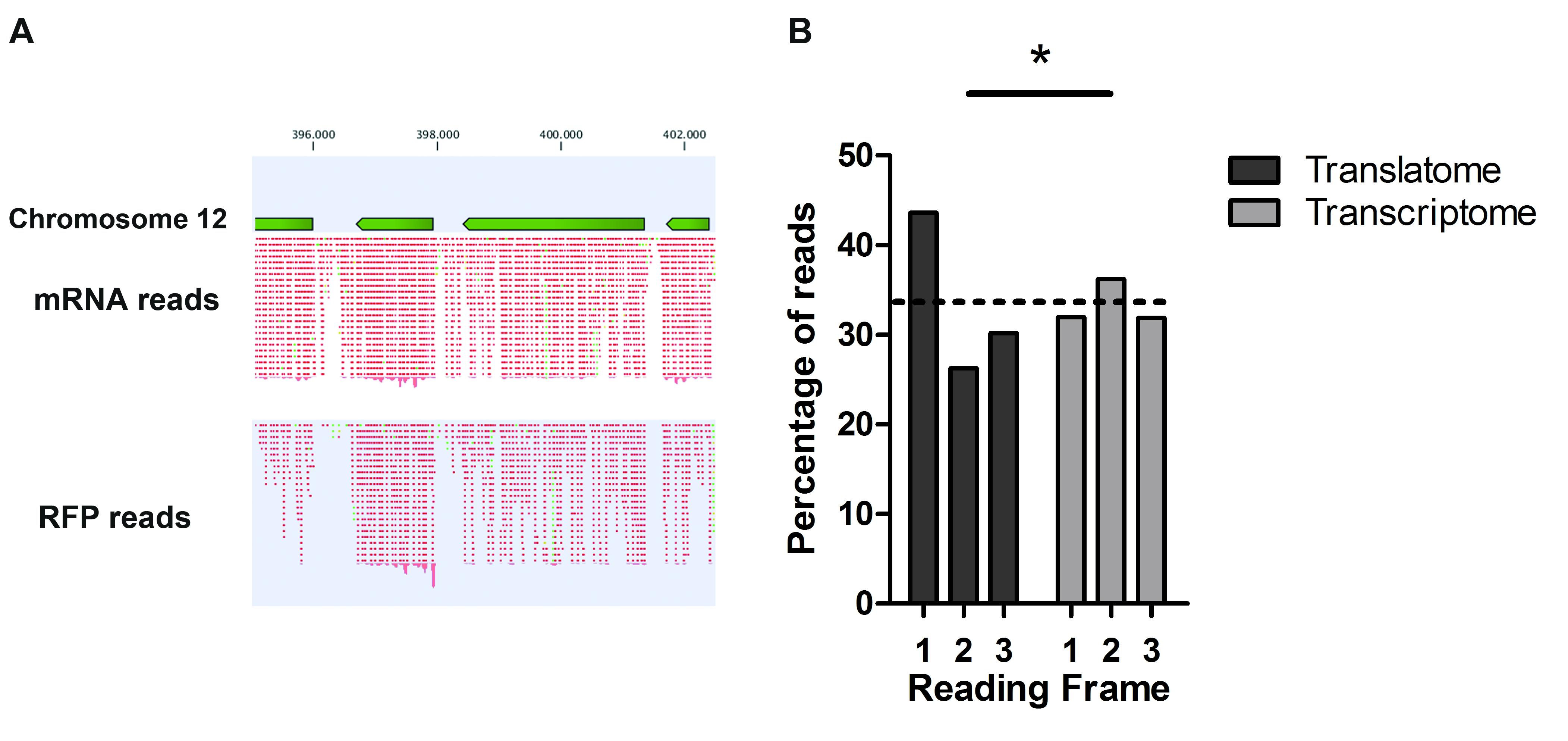

Supplement: Additional file 3: — Ribosome footprints originate from translation activity. (A) Mapping characteristics of the reads obtained in the transcriptome (upper panel) and in the translatome studies (lower panel). A fragment of chromosome 12 (from approx. 395,000 to 400,000bp) is shown. CDSs in the region are represented as green arrows. (B) Mapping periodicity in T. cruzi epimastigotes. Bars represent the percentage of the reads that have their 5´ end mapping to each reading frame (see Materials and Methods). Translatome (dark grey) and transcriptome (light grey) mapping periodicity are shown. [file 12864_2015_1563_MOESM3_ESM.tiff]

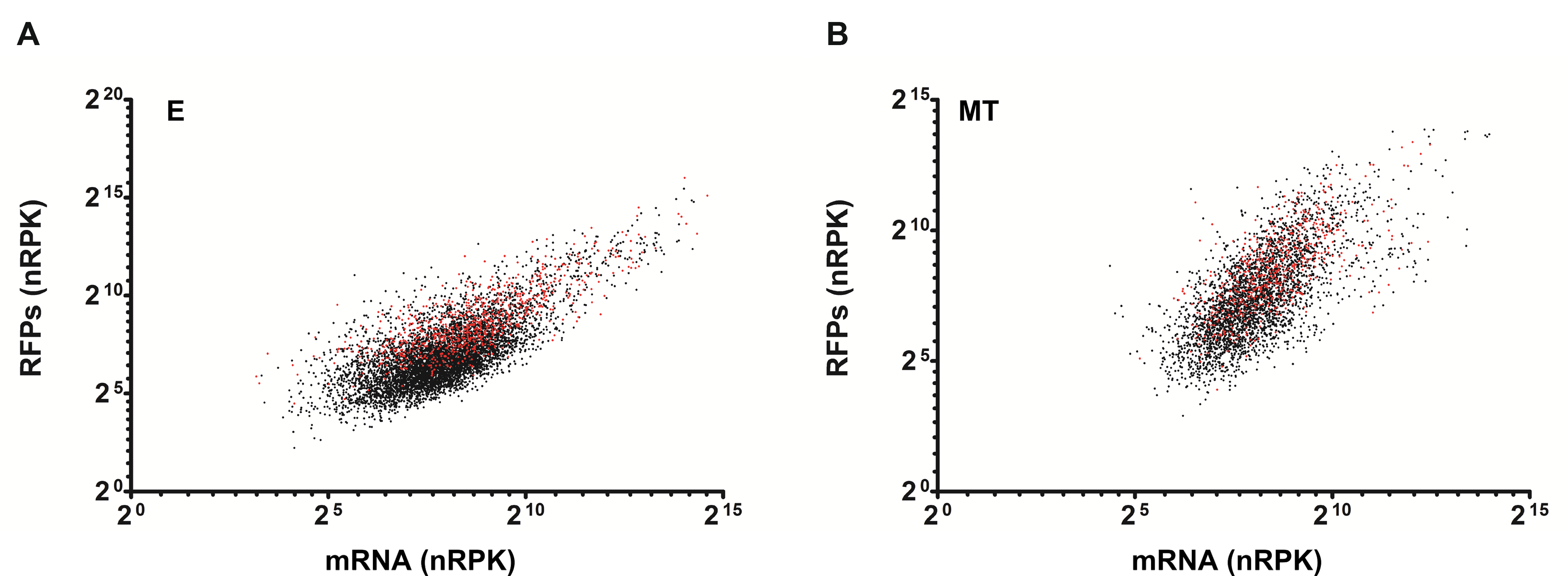

Supplement: Additional file 6: — Transcriptome-translatome correlations. Log-log scatter plot of the estimated expression levels as nRPK. The subset of genes detected in the proteomic studies are shown in red. (A) E: epimastigotes. Pearson correlations of 0.78 and 0.76 were calculated for all genes and for the proteome detected genes respectively. (B) MT: metacyclic trypomastigote. Pearson correlations of 0.66 and 0.63 were calculated for all genes and for the proteome detected genes respectively. [file 12864_2015_1563_MOESM6_ESM.tiff]

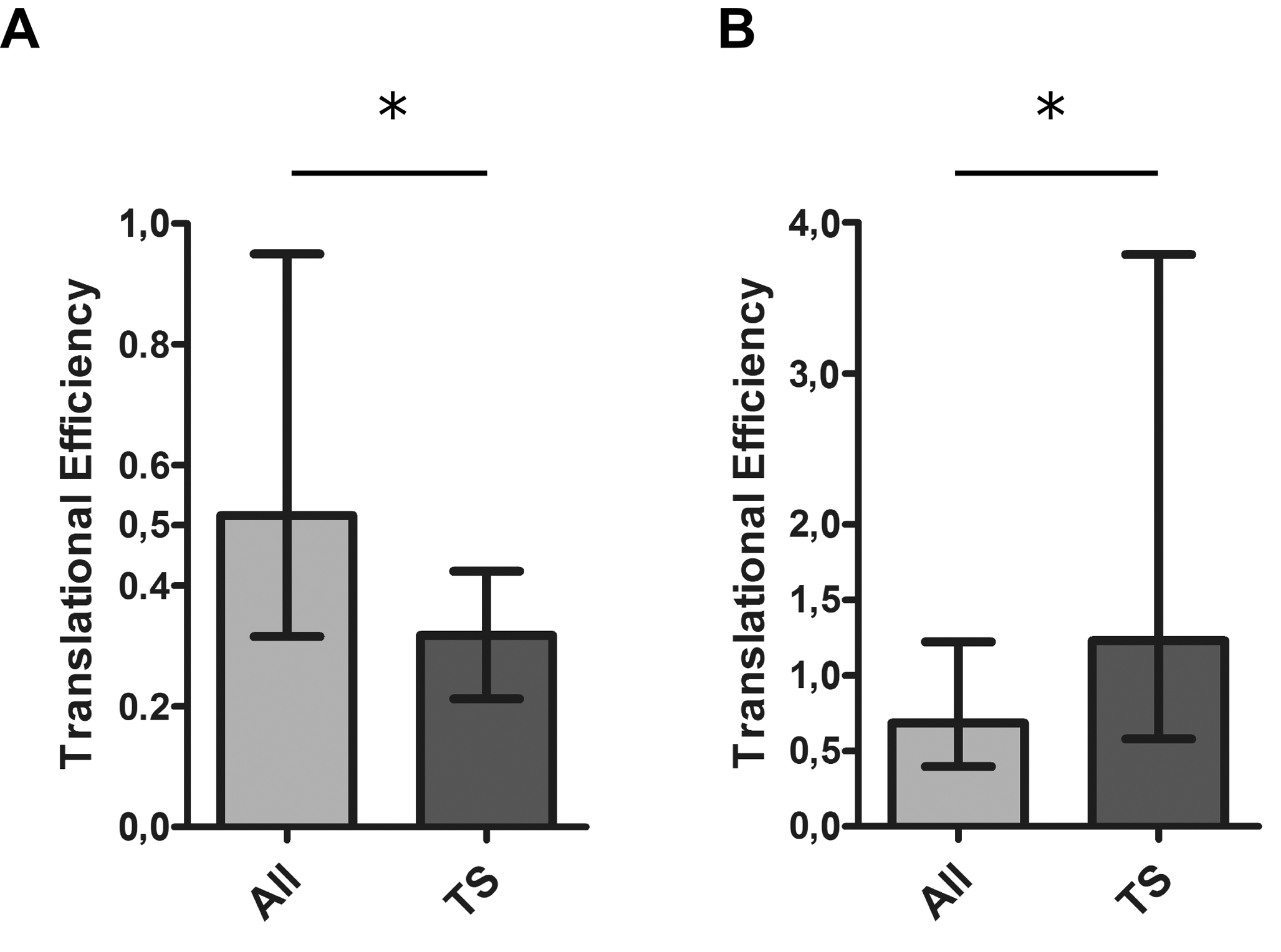

Supplement: Additional file 10: — Trans-sialidase (TS) family gene expression profile in T. cruzi epimastigotes (E) and metacyclic trypomastigotes (MT). Comparison of translational efficiency for the TS family genes with the rest of the genes for the E (A) and MT (B) stages. Y-axis scales in figures A and B are different. [file 12864_2015_1563_MOESM10_ESM.tiff]

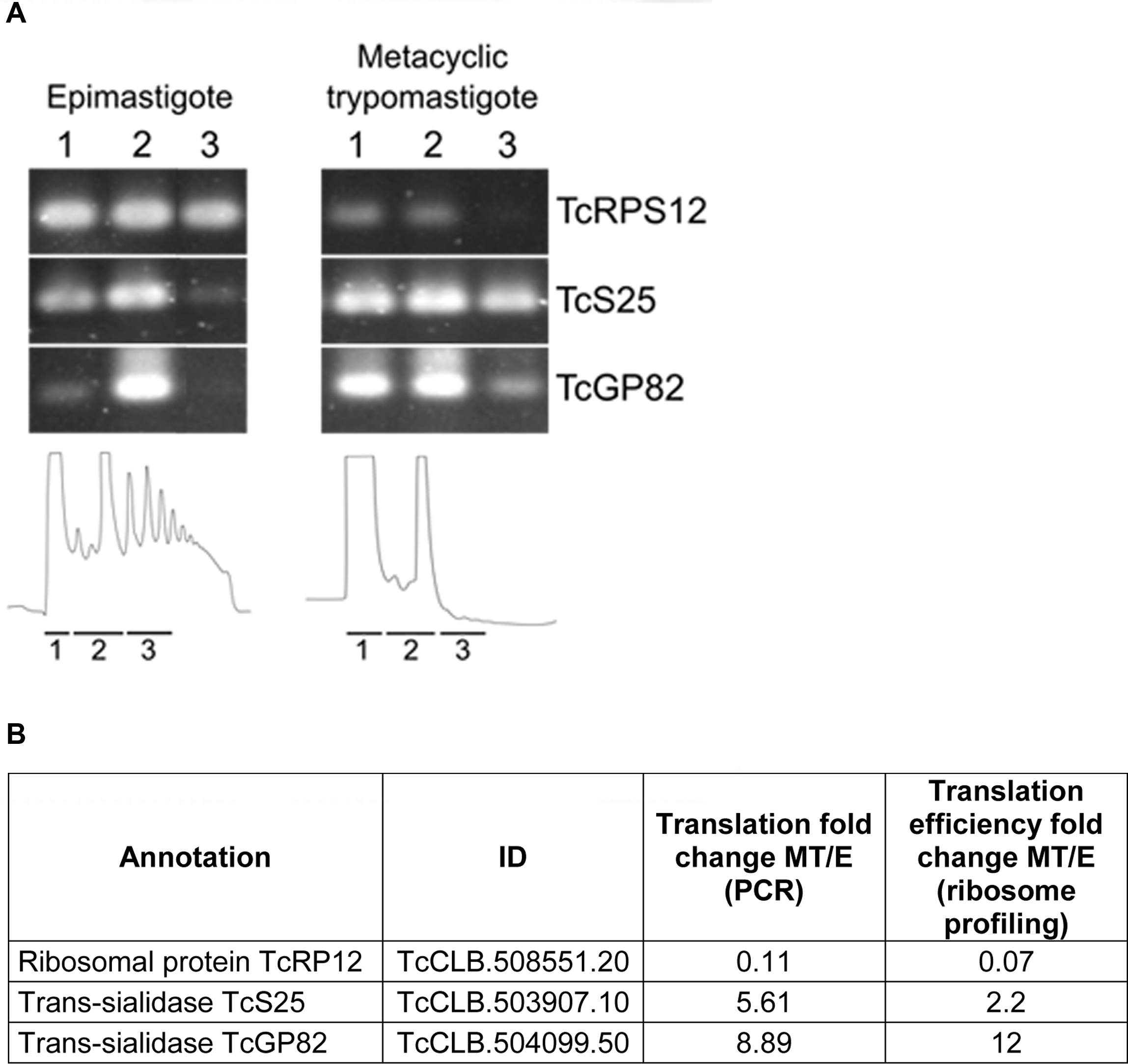

Supplement: Additional file 14: — Independent experimental verification of gene expression levels. (A) Upper panel: result of RT-PCR experiments for ribosome-free mRNA (lane 1), monosomal (lane 2) and polysomal fractions (lane 3) for the selected genes. Lower panel: Polisome profiles obtained by sucrose gradients separation for each stage. The image indicates the span of the 3 fractions analyzed using horizontal lines. (B) Table showing the fold change in translation efficiency (metacyclic trypomastigote divided by epimastigote values) as assessed by both the above RT-PCR experiments (column 3) and by ribosome profiling (column 4). Translation efficiency for the RT-PCR experiments was calculated by first quantifying band density (ImageJ) and then dividing the value obtained in the polysome fraction by the average value obtained in free mRNA and monosome fractions. [file 12864_2015_1563_MOESM14_ESM.tiff]
