# Supplementary material for: Ribosome profiling reveals translation control as a key mechanism generating differential gene expression in Trypanosoma cruzi
Source: BMC Genomics. 2015 Jun 9;16(1):443. doi: 10.1186/s12864-015-1563-8 (PMC4460968; doi:10.1186/s12864-015-1563-8)
Supplement: Additional file 2: — Number of genes present in T. cruzi transcriptome and translatome and their inter stage variation. [file 12864_2015_1563_MOESM2_ESM.docx]

| **Number of genes present in *T. cruzi* transcriptome and translatome and their inter stage variation** | | | | | | |
| --- | --- | --- | --- | --- | --- | --- |
|  | **E*** | **MT*** | **Intersection** | **Up regulated^#^** | **Down regulated^#^** | **Overall regulated**  **(% out of 10600)** |
| **Transcriptome** | 9122 | 9092 | 8876 | 1323 | 1421 | 2744 (25.9%) |
| **Translatome** | 7873 | 6178 | 5652 | 961 | 1134 | 2095 (19.8%) |

*Number of detected genes in each stage (see Materials and Methods for detection criteria).

**^#^**Genes with expression fold change > 2 and a FDR p-value (Benjamini-Hochberg) < 0.05
