# Supplementary material for: Ribosome profiling reveals translation control as a key mechanism generating differential gene expression in Trypanosoma cruzi
Source: BMC Genomics. 2015 Jun 9;16(1):443. doi: 10.1186/s12864-015-1563-8 (PMC4460968; doi:10.1186/s12864-015-1563-8)
Supplement: Additional file 7: — DAVID functional annotation clustering result for genes having a FC>2 (A) and a FC<0.5 (B) in their TE after differentiation. [file 12864_2015_1563_MOESM7_ESM.docx]

**DAVID functional annotation clustering result for genes having a FC>2 (A) and a FC<2 (B) in their TE after differentiation**

**A**

| **Annotation Cluster 1** | **Enrichment Score: 2.85** |  |  |
| --- | --- | --- | --- |
| **Category** | **Term** | **p-value** | **Benjamini** |
| INTERPRO | IPR008377:Trypanosome sialidase | 3.32E-04 | 0.11445359 |
| INTERPRO | IPR013320:Concanavalin A-like lectin/glucanase, subgroup | 8.17E-04 | 0.13895577 |
| PIR_SUPERFAMILY | PIRSF002728:trans-sialidase, trypomastigote type | 0.0012171 | 0.07610747 |
| GOTERM_BP_FAT | GO:0009405~pathogenesis | 0.00239669 | 0.16803629 |
| GOTERM_MF_FAT | GO:0016997~alpha-sialidase activity | 0.00325542 | 0.51513351 |
| GOTERM_MF_FAT | GO:0004308~exo-alpha-sialidase activity | 0.00325542 | 0.51513351 |
|  |  |  |  |
| **Annotation Cluster 2** | **Enrichment Score: 1.30** |  |  |
| **Category** | **Term** | **p-value** | **Benjamini** |
| GOTERM_MF_FAT | GO:0048037~cofactor binding | 0.01102038 | 0.70772278 |
| INTERPRO | IPR015421:Pyridoxal phosphate-dependent transferase, major region, subdomain 1 | 0.02656754 | 0.75534077 |
| SP_PIR_KEYWORDS | pyridoxal phosphate | 0.02719157 | 0.41179862 |
| GOTERM_MF_FAT | GO:0070279~vitamin B6 binding | 0.05805064 | 0.96381493 |
| GOTERM_MF_FAT | GO:0030170~pyridoxal phosphate binding | 0.05805064 | 0.96381493 |
| GOTERM_MF_FAT | GO:0019842~vitamin binding | 0.09139862 | 0.98581666 |
| SP_PIR_KEYWORDS | lyase | 0.32256778 | 0.88257457 |

**B**

| **Annotation Cluster 1** | **Enrichment Score: 9.21** |  |  |
| --- | --- | --- | --- |
| **Category** | **Term** | **p-value** | **Benjamini** |
| GOTERM_BP_FAT | GO:0006412~translation | 2.70E-13 | 7.21E-11 |
| GOTERM_MF_FAT | GO:0003735~structural constituent of ribosome | 2.71E-13 | 6.35E-11 |
| GOTERM_MF_FAT | GO:0005198~structural molecule activity | 7.59E-13 | 8.88E-11 |
| SP_PIR_KEYWORDS | ribosomal protein | 2.33E-12 | 1.77E-10 |
| GOTERM_CC_FAT | GO:0005840~ribosome | 2.13E-09 | 2.39E-07 |
| KEGG_PATHWAY | tcr03010:Ribosome | 4.52E-08 | 1.85E-06 |
| GOTERM_CC_FAT | GO:0030529~ribonucleoprotein complex | 4.78E-08 | 2.67E-06 |
| GOTERM_CC_FAT | GO:0043228~non-membrane-bounded organelle | 4.65E-06 | 1.74E-04 |
| GOTERM_CC_FAT | GO:0043232~intracellular non-membrane-bounded organelle | 4.65E-06 | 1.74E-04 |
|  |  |  |  |
| **Annotation Cluster 2** | **Enrichment Score: 2.20** |  |  |
| **Category** | **Term** | **p-value** | **Benjamini** |
| INTERPRO | IPR015943:WD40/YVTN repeat-like | 9.00E-04 | 0.22278765 |
| INTERPRO | IPR001680:WD40 repeat | 0.0031762 | 0.44779436 |
| INTERPRO | IPR019781:WD40 repeat, subgroup | 0.00409937 | 0.43734639 |
| INTERPRO | IPR017986:WD40 repeat, region | 0.00826057 | 0.53892203 |
| INTERPRO | IPR019782:WD40 repeat 2 | 0.00960281 | 0.49107011 |
| SMART | SM00320:WD40 | 0.01369064 | 0.69859905 |
| SP_PIR_KEYWORDS | wd repeat | 0.0316172 | 0.45688186 |
