# Supplementary material for: Ribosome profiling reveals translation control as a key mechanism generating differential gene expression in Trypanosoma cruzi
Source: BMC Genomics. 2015 Jun 9;16(1):443. doi: 10.1186/s12864-015-1563-8 (PMC4460968; doi:10.1186/s12864-015-1563-8)
Supplement: Additional file 8: — DAVID functional annotation clustering result for the genes with the highest translational efficiency in T. cruzi metacyclic trypomastigotes (MT). [file 12864_2015_1563_MOESM8_ESM.docx]

**DAVID functional annotation clustering result for the genes with the highest translational efficiency in *T. cruzi* metacyclic trypomastigotes (MT)**

| **Annotation Cluster 1** | | **Enrichment Score: 2.01** | |  | |  |
| --- | --- | --- | --- | --- | --- | --- |
| **Category** | | **Term** | | **p-value** | | **Benjamini** |
| INTERPRO | | IPR008377:Trypanosome sialidase | | 8.70E-04 | | 0.01640662 |
| GOTERM_BP_FAT | | GO:0009405~pathogenesis | | 0.00383003 | | 0.06674141 |
| GOTERM_MF_FAT | | GO:0004308~exo-alpha-sialidase activity | | 0.00651465 | | 0.17266287 |
| GOTERM_MF_FAT | GO:0016997~alpha-sialidase activity | | 0.00651465 | | 0.17266287 | |
| INTERPRO | IPR013320:Concanavalin A-like lectin/glucanase, subgroup | | 0.04510088 | | 0.35494585 | |
| PIR_SUPERFAMILY | PIRSF002728:trans-sialidase, trypomastigote type | | 0.13795312 | | 0.35939156 | |
