# Supplementary material for: Ribosome profiling reveals translation control as a key mechanism generating differential gene expression in Trypanosoma cruzi
Source: BMC Genomics. 2015 Jun 9;16(1):443. doi: 10.1186/s12864-015-1563-8 (PMC4460968; doi:10.1186/s12864-015-1563-8)
Supplement: Additional file 9: — DAVID functional annotation clustering result for the genes increasing their translation after T. cruzi epimastigote to metacyclic trypomastigote differentiation. [file 12864_2015_1563_MOESM9_ESM.docx]

**DAVID functional annotation clustering result for the genes increasing their translation after *T. cruzi* epimastigote to metacyclic trypomastigote differentiation**

**A. Genes that do not change their mRNA steady state levels (0.5<FC<2).**

| **Annotation Cluster 1** | **Enrichment Score: 2.41** |  |  |
| --- | --- | --- | --- |
| **Category** | **Term** | **p-value** | **Benjamini** |
| PIR_SUPERFAMILY | PIRSF001204:leishmanolysin | 7.26E-06 | 1.31E-04 |
| INTERPRO | IPR001577:Peptidase M8, leishmanolysin | 2.97E-04 | 0.03535049 |
| GOTERM_BP_FAT | GO:0007155~cell adhesión | 6.63E-04 | 0.07468662 |
| GOTERM_BP_FAT | GO:0022610~biological adhesión | 6.63E-04 | 0.07468662 |
| GOTERM_MF_FAT | GO:0004175~endopeptidase activity | 8.05E-04 | 0.0654341 |
| GOTERM_MF_FAT | GO:0004222~metalloendopeptidase activity | 0.00115694 | 0.04745667 |
| SP_PIR_KEYWORDS | Protease | 0.00186561 | 0.06675913 |
| GOTERM_MF_FAT | GO:0008237~metallopeptidase activity | 0.00397045 | 0.10541373 |
| GOTERM_MF_FAT | GO:0070011~peptidase activity, acting on L-amino acid peptides | 0.00607829 | 0.12017609 |
| GOTERM_MF_FAT | GO:0008233~peptidase activity | 0.00954927 | 0.14887704 |
| GOTERM_BP_FAT | GO:0006508~proteolysis | 0.01104351 | 0.47776583 |
| GOTERM_MF_FAT | GO:0008270~zinc ion binding | 0.03476674 | 0.34598554 |
| GOTERM_MF_FAT | GO:0046914~transition metal ion binding | 0.05969669 | 0.47602132 |
| GOTERM_MF_FAT | GO:0043169~cation binding | 0.07239045 | 0.50408342 |
| GOTERM_MF_FAT | GO:0046872~metal ion binding | 0.07239045 | 0.50408342 |
| GOTERM_MF_FAT | GO:0043167~ion binding | 0.07239045 | 0.50408342 |
|  |  |  |  |
| **Annotation Cluster 2** | **Enrichment Score: 1.90** |  |  |
| **Category** | **Term** | **p-value** | **Benjamini** |
| INTERPRO | IPR008377:Trypanosome sialidase | 0.00905726 | 0.42331585 |
| INTERPRO | IPR013320:Concanavalin A-like lectin/glucanase, subgroup | 0.0096443 | 0.32353511 |
| GOTERM_MF_FAT | GO:0004308~exo-alpha-sialidase activity | 0.0125793 | 0.16241054 |
| GOTERM_MF_FAT | GO:0016997~alpha-sialidase activity | 0.0125793 | 0.16241054 |
| PIR_SUPERFAMILY | PIRSF002728:trans-sialidase, trypomastigote type | 0.02259855 | 0.18593928 |
| GOTERM_BP_FAT | GO:0009405~pathogenesis | 0.02359677 | 0.60596115 |

**B. Results for all the genes (independent of the behavior of the mRNA levels)**

| **Annotation Cluster 1** | **Enrichment Score: 21.83** |  |  |
| --- | --- | --- | --- |
| **Category** | **Term** | **p-value** | **Benjamini** |
| PIR_SUPERFAMILY | PIRSF002728:trans-sialidase, trypomastigote type | 4.54E-25 | 1.86E-23 |
| INTERPRO | IPR008377:Trypanosome sialidase | 1.36E-24 | 4.97E-22 |
| INTERPRO | IPR013320:Concanavalin A-like lectin/glucanase, subgroup | 2.03E-22 | 3.72E-20 |
| GOTERM_MF_FAT | GO:0004308~exo-alpha-sialidase activity | 4.41E-21 | 9.39E-19 |
| GOTERM_MF_FAT | GO:0016997~alpha-sialidase activity | 4.41E-21 | 9.39E-19 |
| GOTERM_BP_FAT | GO:0009405~pathogenesis | 4.56E-21 | 1.24E-18 |
|  |  |  |  |
| **Annotation Cluster 2** | **Enrichment Score: 4.09** |  |  |
| **Category** | **Term** | **p-value** | **Benjamini** |
| INTERPRO | IPR017442:Serine/threonine protein kinase-related | 1.45E-10 | 1.77E-08 |
| SP_PIR_KEYWORDS | kinase | 1.62E-10 | 8.91E-09 |
| INTERPRO | IPR000719:Protein kinase, core | 2.42E-10 | 2.22E-08 |
| INTERPRO | IPR008271:Serine/threonine protein kinase, active site | 1.01E-08 | 7.41E-07 |
| GOTERM_MF_FAT | GO:0004672~protein kinase activity | 7.52E-08 | 8.01E-06 |
| GOTERM_BP_FAT | GO:0006468~protein amino acid phosphorylation | 1.32E-07 | 1.80E-05 |
| GOTERM_MF_FAT | GO:0004674~protein serine/threonine kinase activity | 2.98E-07 | 2.11E-05 |
| SP_PIR_KEYWORDS | serine/threonine-protein kinase | 3.69E-07 | 1.01E-05 |
| INTERPRO | IPR017441:Protein kinase, ATP binding site | 1.20E-06 | 7.35E-05 |
| GOTERM_BP_FAT | GO:0016310~phosphorylation | 1.40E-05 | 0.00126741 |
| GOTERM_BP_FAT | GO:0006793~phosphorus metabolic process | 1.95E-05 | 0.00132621 |
| GOTERM_BP_FAT | GO:0006796~phosphate metabolic process | 1.95E-05 | 0.00132621 |
| INTERPRO | IPR002290:Serine/threonine protein kinase | 7.39E-04 | 0.03801279 |
| SP_PIR_KEYWORDS | atp-binding | 0.00244455 | 0.04387973 |
| SMART | SM00220:S_TKc | 0.00335862 | 0.23339009 |
| SP_PIR_KEYWORDS | nucleotide-binding | 0.02261815 | 0.1891837 |
| GOTERM_MF_FAT | GO:0005524~ATP binding | 0.05420363 | 0.94856906 |
| GOTERM_MF_FAT | GO:0032559~adenyl ribonucleotide binding | 0.05596593 | 0.9140047 |
| GOTERM_MF_FAT | GO:0001883~purine nucleoside binding | 0.09803928 | 0.97434567 |
| GOTERM_MF_FAT | GO:0030554~adenyl nucleotide binding | 0.09803928 | 0.97434567 |
| GOTERM_MF_FAT | GO:0001882~nucleoside binding | 0.10772281 | 0.96882695 |
| GOTERM_MF_FAT | GO:0000166~nucleotide binding | 0.18342689 | 0.98023464 |
| GOTERM_MF_FAT | GO:0032555~purine ribonucleotide binding | 0.25130372 | 0.97338515 |
| GOTERM_MF_FAT | GO:0032553~ribonucleotide binding | 0.25130372 | 0.97338515 |
| GOTERM_MF_FAT | GO:0017076~purine nucleotide binding | 0.34867957 | 0.98960287 |
|  |  |  |  |
| **Annotation Cluster 3** | **Enrichment Score: 1.43** |  |  |
| **Category** | **Term** | **p-value** | **Benjamini** |
| GOTERM_CC_FAT | GO:0005874~microtubule | 3.42E-04 | 0.01528668 |
| INTERPRO | IPR019821:Kinesin, motor region, conserved site | 0.00245261 | 0.10653814 |
| GOTERM_CC_FAT | GO:0015630~microtubule cytoskeleton | 0.00271301 | 0.05929494 |
| GOTERM_CC_FAT | GO:0005856~cytoskeleton | 0.00766752 | 0.10904027 |
| GOTERM_CC_FAT | GO:0044430~cytoskeletal part | 0.00796641 | 0.08605143 |
| SP_PIR_KEYWORDS | motor protein | 0.01943401 | 0.23650473 |
| SP_PIR_KEYWORDS | microtubule | 0.02210082 | 0.21794932 |
| INTERPRO | IPR001752:Kinesin, motor region | 0.02979753 | 0.57428911 |
| SMART | SM00129:KISc | 0.06609935 | 0.83483506 |
| GOTERM_BP_FAT | GO:0007018~microtubule-based movement | 0.18933866 | 0.99992631 |
| GOTERM_MF_FAT | GO:0003777~microtubule motor activity | 0.19562366 | 0.97901532 |
| GOTERM_MF_FAT | GO:0003774~motor activity | 0.31293129 | 0.98821937 |
| GOTERM_BP_FAT | GO:0007017~microtubule-based process | 0.33631689 | 0.99999584 |
| GOTERM_CC_FAT | GO:0043228~non-membrane-bounded organelle | 0.91384479 | 0.99999986 |
| GOTERM_CC_FAT | GO:0043232~intracellular non-membrane-bounded organelle | 0.91384479 | 0.99999986 |
|  |  |  |  |
| **Annotation Cluster 4** | **Enrichment Score: 1.42** |  |  |
| **Category** | **Term** | **p-value** | **Benjamini** |
| INTERPRO | IPR015903:Ribonucleoprotein, BRUNO-like | 0.00456421 | 0.17017843 |
| INTERPRO | IPR012677:Nucleotide-binding, alpha-beta plait | 0.04682143 | 0.66710302 |
| INTERPRO | IPR000504:RNA recognition motif, RNP-1 | 0.06897573 | 0.73057716 |
| SMART | SM00360:RRM | 0.14565658 | 0.87415741 |
