# Supplementary material for: Ribosome profiling reveals translation control as a key mechanism generating differential gene expression in Trypanosoma cruzi
Source: BMC Genomics. 2015 Jun 9;16(1):443. doi: 10.1186/s12864-015-1563-8 (PMC4460968; doi:10.1186/s12864-015-1563-8)
Supplement: Additional file 12: — DAVID functional annotation clustering result for the genes decreasing their translation after T. cruzi epimastigote to metacyclic trypomastigote differentiation. [file 12864_2015_1563_MOESM12_ESM.docx]

**DAVID functional annotation clustering result for the genes decreasing their translation after *T. cruzi* epimastigote to metacyclic trypomastigote differentiation**

**A. Genes that do not change their mRNA steady state levels (0.5<FC<2).**

| **Annotation Cluster 1** | **Enrichment Score: 7.01** |  |  |
| --- | --- | --- | --- |
| **Category** | **Term** | **p-value** | **Benjamini** |
| GOTERM_MF_FAT | GO:0003735~structural constituent of ribosome | 2.10E-12 | 3.76E-10 |
| GOTERM_BP_FAT | GO:0006412~translation | 1.23E-11 | 3.19E-09 |
| GOTERM_MF_FAT | GO:0005198~structural molecule activity | 1.52E-11 | 1.36E-09 |
| SP_PIR_KEYWORDS | ribosomal protein | 8.65E-11 | 4.76E-09 |
| KEGG_PATHWAY | tcr03010:Ribosome | 1.03E-07 | 3.49E-06 |
| GOTERM_CC_FAT | GO:0005840~ribosome | 1.34E-06 | 8.19E-05 |
| GOTERM_CC_FAT | GO:0030529~ribonucleoprotein complex | 9.56E-06 | 2.91E-04 |
| SP_PIR_KEYWORDS | ribonucleoprotein | 0.00120093 | 0.03250542 |
| GOTERM_CC_FAT | GO:0043228~non-membrane-bounded organelle | 0.00128615 | 0.02582904 |
| GOTERM_CC_FAT | GO:0043232~intracellular non-membrane-bounded organelle | 0.00128615 | 0.02582904 |
|  |  |  |  |
| **Annotation Cluster 2** | **Enrichment Score: 1.90** |  |  |
| **Category** | **Term** | **p-value** | **Benjamini** |
| INTERPRO | IPR019955:Ubiquitin supergroup | 0.00951977 | 0.97484428 |
| SMART | SM00213:UBQ | 0.01050842 | 0.38488445 |
| INTERPRO | IPR000626:Ubiquitin | 0.01982029 | 0.97879935 |
|  |  |  |  |
| **Annotation Cluster 3** | **Enrichment Score: 1.17** |  |  |
| **Category** | **Term** | **p-value** | **Benjamini** |
| GOTERM_MF_FAT | GO:0016866~intramolecular transferase activity | 0.03305563 | 0.86542778 |
| GOTERM_MF_FAT | GO:0009982~pseudouridine synthase activity | 0.06897136 | 0.92257578 |
| GOTERM_BP_FAT | GO:0009451~RNA modification | 0.07555413 | 0.96633199 |
| GOTERM_BP_FAT | GO:0001522~pseudouridine synthesis | 0.08512142 | 0.94387685 |
| GOTERM_MF_FAT | GO:0003723~RNA binding | 0.09657679 | 0.95168424 |

**B. Results for all the genes (independent of the behavior of the mRNA levels)**

| **Annotation Cluster 1** | **Enrichment Score: 23.38** |  |  |
| --- | --- | --- | --- |
| **Category** | **Term** | **p-value** | **Benjamini** |
| GOTERM_MF_FAT | GO:0003735~structural constituent of ribosome | 1.18E-36 | 4.18E-34 |
| GOTERM_MF_FAT | GO:0005198~structural molecule activity | 1.61E-34 | 2.84E-32 |
| SP_PIR_KEYWORDS | ribosomal protein | 2.20E-33 | 2.25E-31 |
| GOTERM_BP_FAT | GO:0006412~translation | 1.78E-27 | 8.06E-25 |
| GOTERM_CC_FAT | GO:0005840~ribosome | 1.47E-24 | 1.25E-22 |
| GOTERM_CC_FAT | GO:0030529~ribonucleoprotein complex | 3.81E-20 | 1.62E-18 |
| KEGG_PATHWAY | tcr03010:Ribosome | 3.75E-18 | 2.59E-16 |
| GOTERM_CC_FAT | GO:0043232~intracellular non-membrane-bounded organelle | 5.15E-11 | 1.46E-09 |
| GOTERM_CC_FAT | GO:0043228~non-membrane-bounded organelle | 5.15E-11 | 1.46E-09 |
|  |  |  |  |
| **Annotation Cluster 2** | **Enrichment Score: 1.98** |  |  |
| **Category** | **Term** | **p-value** | **Benjamini** |
| GOTERM_BP_FAT | GO:0008612~peptidyl-lysine modification to hypusine | 0.00474043 | 0.51203244 |
| GOTERM_BP_FAT | GO:0018205~peptidyl-lysine modification | 0.00474043 | 0.51203244 |
| GOTERM_BP_FAT | GO:0046516~hypusine metabolic process | 0.00474043 | 0.51203244 |
| GOTERM_BP_FAT | GO:0006575~cellular amino acid derivative metabolic process | 0.11751106 | 0.88673689 |
|  |  |  |  |
| **Annotation Cluster 3** | **Enrichment Score: 1.78** |  |  |
| **Category** | **Term** | **p-value** | **Benjamini** |
| GOTERM_BP_FAT | GO:0018130~heterocycle biosynthetic process | 0.00477015 | 0.41813231 |
| GOTERM_BP_FAT | GO:0006766~vitamin metabolic process | 0.00718458 | 0.47965995 |
| GOTERM_BP_FAT | GO:0042364~water-soluble vitamin biosynthetic process | 0.00718458 | 0.47965995 |
| GOTERM_BP_FAT | GO:0009110~vitamin biosynthetic process | 0.00718458 | 0.47965995 |
| GOTERM_BP_FAT | GO:0006767~water-soluble vitamin metabolic process | 0.00718458 | 0.47965995 |
| GOTERM_BP_FAT | GO:0044272~sulfur compound biosynthetic process | 0.03119956 | 0.79717206 |
| GOTERM_BP_FAT | GO:0006790~sulfur metabolic process | 0.03119956 | 0.79717206 |
| GOTERM_BP_FAT | GO:0019438~aromatic compound biosynthetic process | 0.04183621 | 0.82794977 |
| GOTERM_BP_FAT | GO:0042723~thiamin and derivative metabolic process | 0.0561501 | 0.84585459 |
| GOTERM_BP_FAT | GO:0042724~thiamin and derivative biosynthetic process | 0.0561501 | 0.84585459 |
|  |  |  |  |
| **Annotation Cluster 4** | **Enrichment Score: 1.74** |  |  |
| **Category** | **Term** | **p-value** | **Benjamini** |
| INTERPRO | IPR014721:Ribosomal protein S5 domain 2-type fold | 7.20E-04 | 0.19132548 |
| INTERPRO | IPR006204:GHMP kinase | 0.04614412 | 0.9598903 |
| INTERPRO | IPR013750:GHMP kinase, C-terminal | 0.17685466 | 0.99786963 |
